# Targeting the kynurenine pathway: a novel approach in tumour therapy

**DOI:** 10.1017/erm.2025.5

**Published:** 2025-03-05

**Authors:** Shuoqi Lin, Genggeng Zheng, Yuxiang Yan, Tesen Liao, Bohua Su, Dali Zheng

**Affiliations:** 1 Fujian Key Laboratory of Oral Diseases and Fujian Provincial Engineering Research Center of Oral Biomaterial and Stomatological Key Laboratory of Fujian College and University, School and Hospital of Stomatology, Fujian Medical University, Fuzhou, People’s Republic of China; 2 Department of Preventive Dentistry, School and Hospital of Stomatology, Fujian Medical University, Fuzhou, People’s Republic of China

**Keywords:** cancer, combinational therapy, kynurenine pathway, precision medicine, targeted therapy

## Abstract

**Background:**

Cancer cells interact with their surroundings to promote tumour formation and metastasis, often requiring a constant supply of amino acids. The reprogramming of tryptophan (Trp) metabolism is highly activated in tumours, providing essential biological raw materials and energy for malignant tumour progression. Among these metabolic pathways, the kynurenine pathway (KP) plays a crucial role, making it a promising target for tumour therapy.

**Methods:**

This study comprehensively examines the roles of KP metabolites in tumour growth and evaluates therapeutic strategies targeting this pathway.

**Results:**

Targeting the KP in Trp metabolism presents new possibilities for tumour treatment. The study highlights various strategies, including traditional inhibition of key enzymes, novel drug delivery systems for enzyme targeting and mechanism-derived combination therapies. These approaches aim to enhance the precision and effectiveness of tumour therapy by modulating KP activity.

**Conclusions:**

A deeper understanding of KP metabolism in tumour progression opens new avenues for therapeutic intervention.

## Graphical abstract

**Figure:**
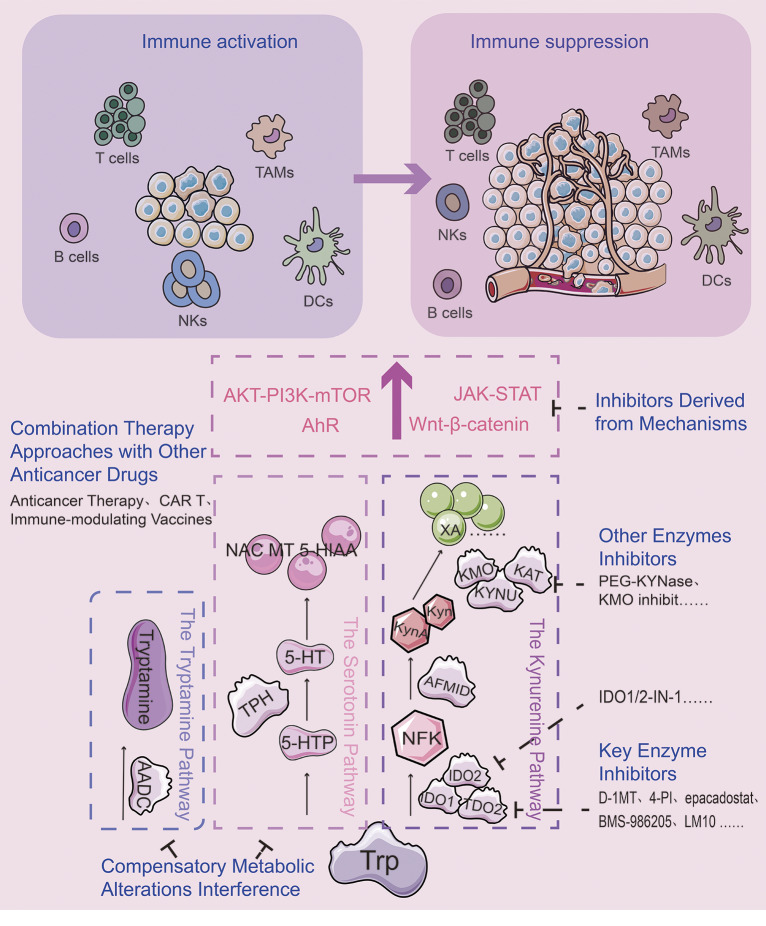
Strategies for targeting the kynurenine pathway. Conventional targeting of key enzymes, novel targeting of key enzyme delivery systems and mechanism-derived targets and combination therapies for tumour therapy targeting the kynurenine pathway.

## Introduction

In the 21st century, cancer is a significant social, public health and economic issue. According to recent global epidemiological reports, approximately 20 million new cases of cancer are expected to be diagnosed worldwide in 2022, with 9.7 million deaths attributed to the disease (Ref. 1). While traditional anti-cancer therapies such as surgery, chemotherapy and radiation have shown progress, the field of cancer treatment is on a quest aimed at improving survival rates (Ref. 2). The emerging therapeutic avenues including immunotherapy, gene therapy and molecularly targeted therapies offer great promise in this context (Ref. 3). A characteristic of cancer is the abnormal control of cellular metabolism, whereby cancer cells must modify the processes that enable them to absorb extracellular metabolites and optimize the activity of these enzymes to adapt and endure extreme environmental stressors (Ref. 4). Consequently, targeting metabolic reprogramming has become a promising frontier in cancer therapy (Ref. 5).

Tryptophan (Trp) metabolic reprogramming is highly active in tumours, with the kynurenine pathway (KP) being the primary metabolic route through which over 95% of tryptophan is degraded into a variety of biologically active compounds. This process is catalyzed by key enzymes, including indoleamine 2,3-dioxygenase 1 (IDO1) and tryptophan 2,3-dioxygenase 2 (TDO2) and the levels of these metabolites are closely associated with the malignant features of the tumour (Refs 6, 7). Studies using inhibitors of key enzymes involved in the metabolism of the kynurenine pathway have shown improved efficacy both in vitro and in vivo (Ref. 8). Among the most extensively studied enzymes targeting this pathway is IDO1. When used in combination with immune checkpoint inhibitors, IDO1 inhibitors have shown potential to enhance anti-tumour immune responses in animal models (Ref. 9). However, a phase III therapeutic trial using the PD-1 checkpoint inhibitor pembrolizumab in conjunction with the IDO1 inhibitor epacadostat to treat malignant melanoma failed (Ref. 10). This raises concerns about the effectiveness of targeting the kynurenine pathway in cancer therapy.

This article aims to clarify a few questions: Targeting the KP in the past has prioritized the inhibition of crucial enzymes while ignoring the regulation of other enzymes. Can other metabolic enzymes be used as therapeutic targets? How should we decide between targeting key enzymes and other metabolic enzymes? Meanwhile, cancer cells interact with their surroundings, leading to tumour formation. So will the newly discovered regulatory effects of the kynurenine pathway metabolites on tumours and the tumour microenvironment (cells as well as infiltrating bacteria) shed new light on drug design? Furthermore, do these modulatory effects provide a theoretical foundation for combination therapies?

## Tryptophan metabolism and its role in tumour progression

### Kynurenine pathway is the principal route of tryptophan catabolism

**Figure 1.**
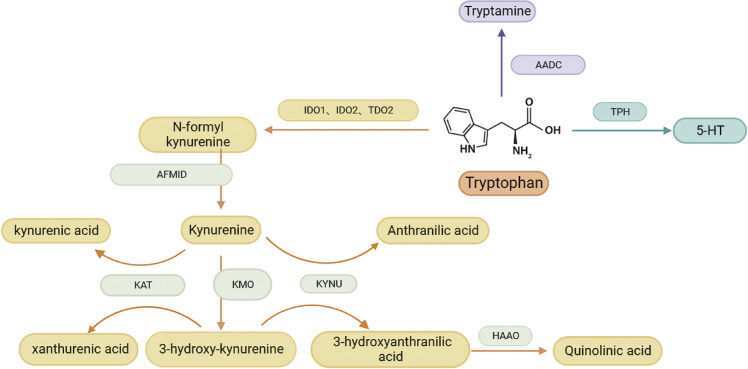
Catabolic pathways of tryptophan. The kynurenine pathway, the tryptamine pathway and the serotonin pathway together constitute the tryptophan catabolic pathway.

In addition to being a necessary amino acid for protein synthesis, L-Trp also contributes to homeostasis by generating a range of metabolites via intricate metabolic processes. A small portion of ingested tryptophan is utilized for anabolic processes, while the majority follows three main catabolic pathways (Figure 1): First, tryptamine is created when aromatic L-amino acid decarboxylase (AADC) decarboxylates it (Ref. 11). Secondly, about 5% of tryptophan forms 5-hydroxytryptamine (5-HT) through tryptophan hydroxylase (TPH) (Ref. 12). Thirdly, three rate-limiting enzymes, IDO1, IDO2 and TDO2, convert approximately 95% of free Trp to N-formyl kynurenine (NFK). Arylformamidase (AFMID) then converts NFK to kynurenine (Kyn and kynurenic aminotransferase (KATI-KATIII) then converts Kyn to kynurenic acid (KA). While kynureninase (KYNU) converts kyn to anthranilic acid (AA). Through Kynurenine 3-monooxygenase (KMO), Kyn also produces 3-hydroxy-kynurenine (3-HK), which is subsequently processed by KYNU to produce 3-hydroxyanthranilic acid (3-HAA). 3-hydroxyanthranilate 3,4-dioxygenase (HAAO) transforms 3-HAA into neurotoxic quinolinic acid (QA), which certain cells can then transform into NAD^+^, a crucial coenzyme in energy metabolism. 3-HK can also be converted by KATI-KATIII catalysis to produce xanthurenic acid (XA) (Ref. 13).

The tryptophan metabolites listed above play a role in the organism’s control of inflammation, immunological response and excitatory neurotransmission (Ref. 14). This article focuses on the role of tryptophan metabolites generated by the KP in tumour growth. The technique known as single-cell RNA sequencing, or scRNA-seq, is used to highlight the diversity of intricate biological systems. Ashley et al. collected samples before Tyrosine kinase inhibitors targeted therapy and residual lesions during progressive disease (PD) for single-cell sequencing analysis, revealing upregulation of KP metabolic pathway enzyme gene expression during PD (Ref. 15). Based on high-throughput data, it is evident that tryptophan metabolism is abnormally activated in various cancers, with KP metabolic pathway activation being particularly pronounced.

### Significance of targeting the kynurenine pathway: the kynurenine pathway metabolites facilitate in carcinogenesis

In various tumours, metabolites and metabolic enzymes of the kynurenine pathway contribute to the malignant progression of tumour proliferation, stemness and migratory invasion to varying degrees (Refs 16, 17, 18). Despite differences in the mechanisms by which metabolites and enzymes act, most of them advance the malignant progression of tumours, revealing an important role for the targeted kynurenine pathway in tumour therapy (Ref. 19). Clarifying the regulatory mechanisms of metabolites and enzymes can help refine tumour treatment strategies (Ref. 20). Therefore, we will later summarize the potential mechanisms of action for various KP metabolites and enzymes.

Increased expression of IDO1 in the intestinal epithelium of colorectal cancer patients promotes tumourigenesis and influences prognosis, likely through the activation of β-catenin by Kyn, a metabolite of the kynurenine pathway. This activation enhances cancer cell proliferation and inhibits apoptosis via the PI3K/AKT signaling pathway (Ref. 16). In colorectal cancer, TDO2 further promotes tumour proliferation by enhancing glycolysis and a reduction in tryptophan levels also diminishes the quality of life for patients (Ref. 21). The prognosis and pathological grading of gliomas are positively correlated with the expression and activity of IDO1 and TDO2. This effect is likely mediated by tryptophan catabolism, which produces Kyn, activating the Aryl hydrocarbon receptor (AhR) and stimulating glioma cell motility and invasion through the downstream aquaporin protein AQP4 (Ref. 22). In melanoma, Kyn and 6-formylbenzo[3,2-b]carbazole (FICZ) have anti-proliferative activity on human melanoma cells. 1 pM L-Kyn significantly inhibits the proliferation of A375 cells, while 5 mM of L-Kyn inhibits DNA synthesis in normal human melanoma cells HEMA, and 50 μM FICZ and 5 mM kynurenic acid KynA both markedly cause apoptosis to A375 cells line (Ref. 23). These findings suggest that different concentrations of KP metabolites may exert distinct roles in tumour biology. KMO is aberrantly expressed in the cell membranes of breast cancer cells and other tumours, promoting migration and invasion (Ref. 24). In HepG2 hepatoma cells, Kyn is a significant byproduct of tryptophan breakdown by TDO2, and its buildup in HepG2 cells may be a key mechanism of tumour immune resistance (Ref. 25). Kyn is a major metabolite of tryptophan degradation by TDO2 in HepG2 hepatoma cells, and its accumulation in HepG2 cells may be an important mechanism of tumour immune resistance (Ref. 26). Moreover, activation of the TDO2-Kyn-AhR pathway facilitates PD-L1-mediated immune evasion and enhances stemness, contributing to liver metastasis from colon cancer (Ref. 27). In cervical cancer, Kyn increased the ability of tumour spheroid formation and the expression of tumour stem cell genes (e.g. *Oct4* and Sox2) (Ref. 28). In non-small cell lung cancer, Kyn can increase the stemness of cells from non-small cell lung cancer by activating AhR via the JAK2/STAT3 signaling pathway (Ref. 29). Additionally, tobacco-derived nitrosamines upregulate IDO1 expression, promoting carcinogenesis in non-small cell lung cancer (Ref. 30). In pancreatic cancer, nitric oxide induces IDO1-Kyn-AhR signaling thereby enhancing the disease aggressiveness (Ref. 31). The conversion of Kyn to 3-HK in Diffuse Large B Cell Lymphoma (DLBCL) is catalyzed by KMO, and the resultant 3-HK may be implicated in controlling DLBCL cell survival through NAD^+^ production (Ref. 32). Furthermore, Kyn has been shown to promote vascular endothelial cell proliferation, potentially supporting tumour blood supply (Ref. 33).

Tryptophan metabolism can provide new ideas for tumour therapy in addition to new horizons for tumour diagnosis. Recent studies, including those by Mandarano et al., have highlighted the potential of tryptophan metabolites as prognostic markers. Specifically, IDO1 catabolic activity can be assessed using the serum Kyn/Trp Ratio (KTR), which has been shown to serve as an independent prognostic factor for various tumour types (Ref. 34). In patients undergoing immunotherapy for several solid tumours, the serum KTR may provide predictive and prognostic insights, reflecting key mechanisms of immune resistance (Ref. 35). Elevated Kyn/Trp levels correlate with advanced tumour stages (II and III), increased density of tumor-infiltrating lymphocytes and a higher likelihood of recurrence, as observed in non-small cell lung cancer (Refs 36, 37). Additionally, IDO1 activity could be a contributing factor and a predictive marker of resistance to anti-PD-1 therapy (Ref. 38). In melanoma, serum KTR also serves as a valuable predictor for early intervention and disease outcomes (Ref. 39). Furthermore, KTR is used as a predictive marker for progression-free survival and cancer-specific survival, as well as a sign of aggressiveness in clear cell renal cell carcinoma (Ref. 40). Although IDO1 is usually less expressed in normal tissues, IDO1 activity was observed in malignant cells, which has the potential to be a diagnostic marker (Ref. 41). The fact that the above metabolites have the potential to be used as tumour diagnostic markers is also a side point to the fact that they are more different in cancerous and normal tissues, which makes tumour therapy targeting the kynurenine pathway safer.

## Tumour therapeutic strategies targeting the Kynurenine pathway

### Direct targeting of key enzymes in tryptophan metabolism

#### Key enzymes in kynurenine pathway

Targeting key enzymes in tryptophan metabolism can effectively reduce the production of downstream unfavorable metabolites, thereby suppressing tumour growth. Tryptophan catabolism can also be divided into the following two types according to the mode of action of the key enzymes of metabolism: either shattering the indole ring to generate kynurenine or retaining it (as in the case of 5-hydroxytryptamine, melatonin and indole pyruvate) (Ref. 42). In mammals, three enzymes – IDO1, IDO2 and TDO2 – catalyze the breakdown of the indole ring, and they are named for their ability to incorporate two oxygen atoms into the product. Each of these enzymes exhibits controlled expression and a distinct tissue specialization. TDO2 is considered to be the main regulator of Trp catabolism and is in charge of controlling Trp contents (Ref. 9), while IDO1 becomes crucial under pathological conditions for modulating Tryptophan metabolism (Ref. 43). Furthermore, the enzymatic activities of these enzymes towards the substrate tryptophan differed greatly, with IDO1 (*K*_m_~20 μmol•L^−1^) > TDO2 (*K*_m_~90 μmol•L^−1^) > IDO2 (*K*_m_~6.8 mmol•L^−1^) (Ref. 44).

**Figure 2.**
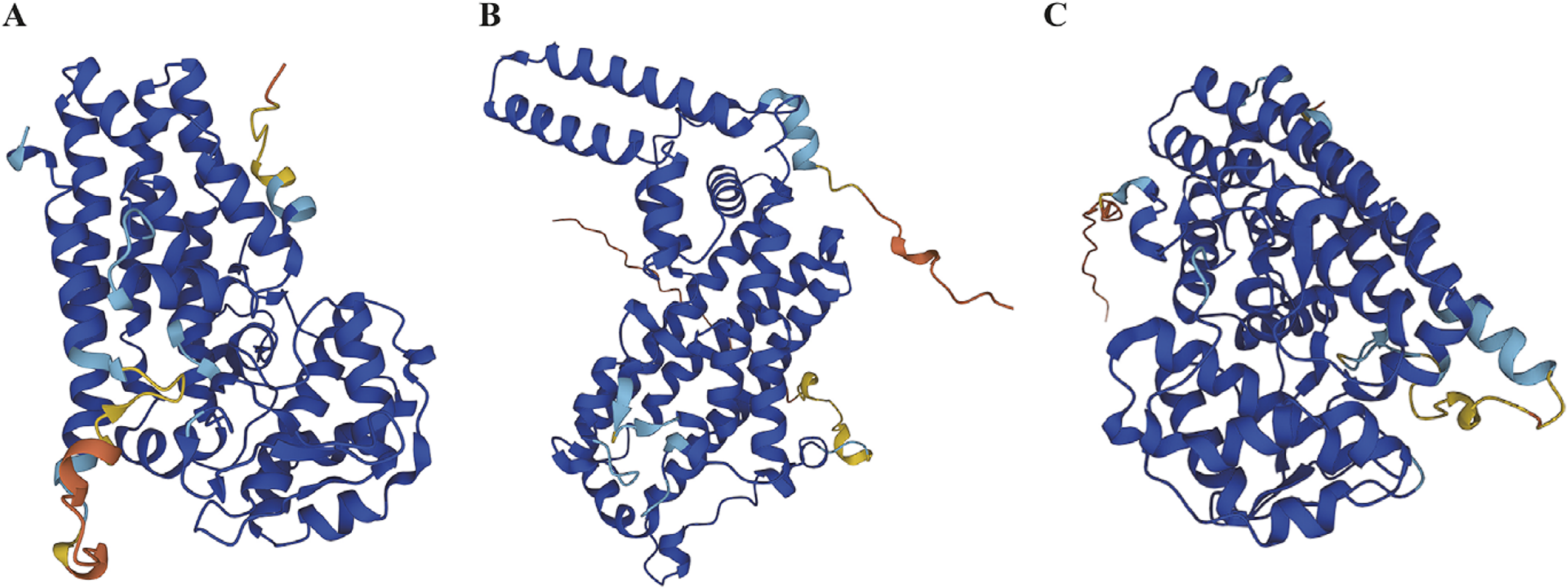
Structure of the major kynurenine pathway metabolizing enzymes. **A**. Structure of indoleamine 2,3 dioxygenase1 (IDO1). **B**. Structure of tryptophan 2,3-dioxygenase 2 (TDO2). (C) Structure of indoleamine 2,3 dioxygenase2 (IDO2).

**Figure 3.**
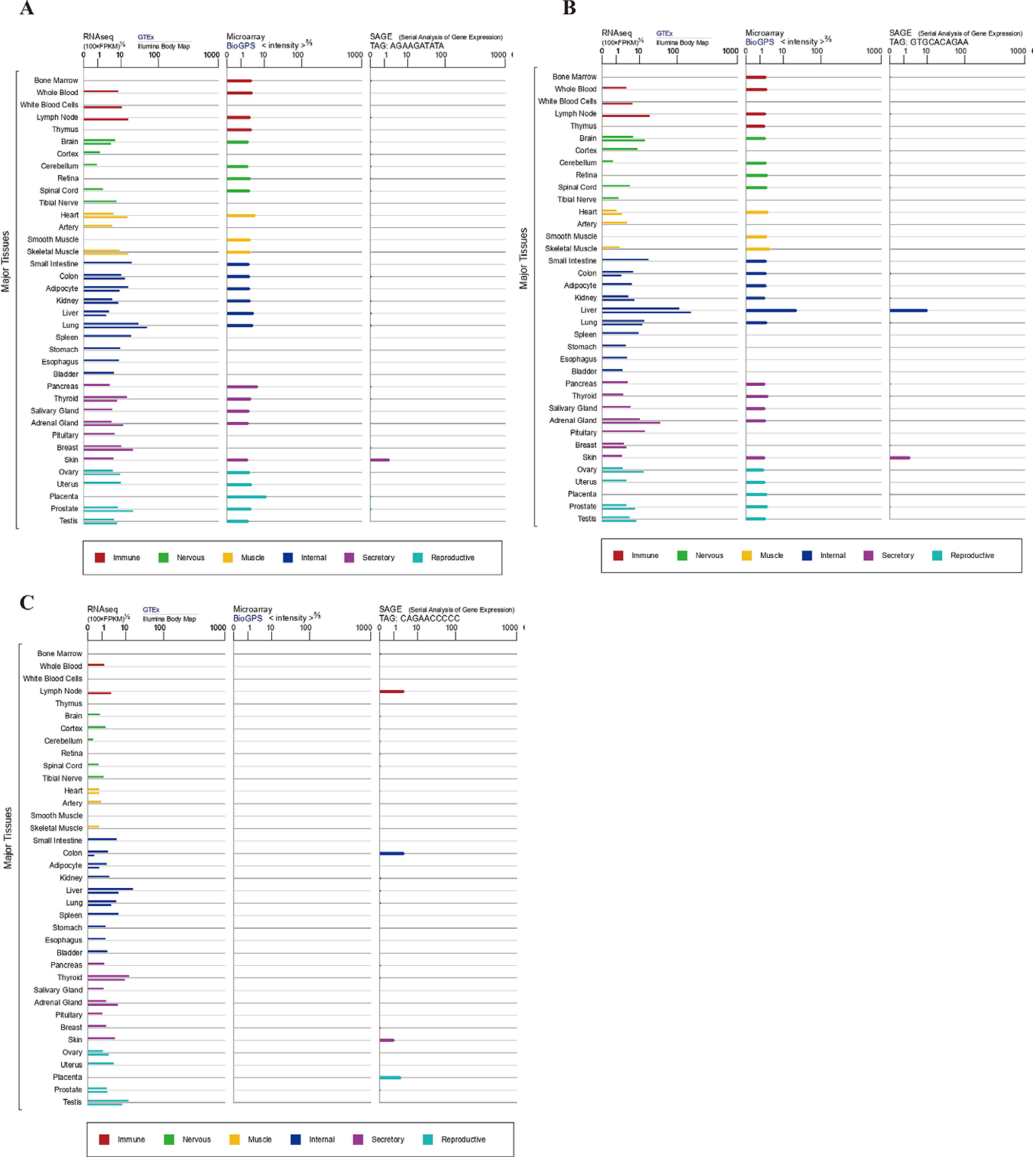
Tissue expression for major metabolic enzymes of the kynurenine pathway. mRNA expression in normal human tissues from GTEx, llumina, BioGPs, and SAGE. **A**. IDO1. **B**. TDO2. (C)IDO2.

IDO1 is a 45 kDa monomeric enzyme containing a heme group, which often crystallizes into a dimeric form (Figure 2A) (Ref. 45). The active site consists of two lipophilic regions: one contains the heme, serving as the primary binding site for tryptophan, while the other forms the entrance to the binding pocket (Ref. 46). Two phosphorylated tyrosine residues, Y115 and Y253, regulate IDO1 activity. Through phosphorylation, these residues can induce conformational changes in IDO1, ultimately reducing its enzymatic activity (Ref. 47). IDO1 can be found in a diverse range of immune cells, including astrocytes, macrophages and dendritic cells (DCs) (Ref. 48). Moreover, it exhibits widespread presence in tumour cells (Figure 3A) (Ref. 49).

TDO2 is a homotetrameric cytosolic enzyme with a molecular mass of 35-45 kDa encoded by the TDO2 gene. Human tryptophan 2,3-dioxygenase 2 (hTDO2) monomers consist of 15 α-helices that can be divided into three major regions: the N-terminal region, the large structural domain and the small structural domain. Three bidirectional axes that are perpendicular to each other connect the four monomers, with stronger interaction between the two connected monomers, in which the two C-shaped dimers are clamped perpendicular to each other to form a tight tetramer (Figure 2B) (Ref. 50). Compared to hIDO1, hTDO2 necessitates a highly specific substrate for binding, with L-Trp being the only relevant native substrate (Ref. 51). TDO2 is predominantly localized within hepatic tissues, with marginal expression detected in adrenal glands, lungs and brain (Figure 3B) (Ref. 52). TDO2 expression can be induced in other tissues, such as the epididymis, placenta, testis, brain and pregnant uterus, in response to regulatory stimuli like glucocorticoids and norepinephrine (Ref. 46).

Clarifying the structure of the key enzyme is beneficial to drug design, and drugs with high selectivity can minimize side effects. Additionally, understanding the distribution of these enzymes across different tissues helps in selecting more targeted inhibitors, tailored to the location and characteristics of the tumour.

#### Traditional key enzyme inhibitors

Based on the relevance of high IDO1 and TDO2 expression in many tumours and their poor prognosis, and the role of many products of the KP metabolic in driving tumour development, we believe it is reasonable to explore IDO inhibitors, TDO2 inhibitors and dual IDO1/TDO2 inhibitors. The expression and activity of IDO1 are influenced by multiple factors and regulated at the transcriptional level through several pathways, including: (1) NF-κB pathway; (2) CCCTC (C: Cytosine, T: Thymine) -binding factor (Ref. 53); and (3) Specific DC response elements bind to AhR and promote Kyn-dependent IDO1 expression (Ref. 54). Currently, no selective TDO2 inhibitors have entered clinical trials, though three dual IDO1/TDO2 inhibitors are being studied. Common IDO1 inhibitors include tryptophan analogs (e.g., D-1MT), aryl imidazoles and their derivatives (e.g., 4-PI), N-hydroxyamidine (e.g., epacadostat), quinones, quinolines and others (e.g., BMS-986205), etc. Among them, D-1MT can indirectly inhibit the KP by reversing tryptophan depletion-induced mTORC1 inhibition in human Teff cells (Refs 55, 56); Epacadostat, a more commonly tested compound in clinical trials, unfortunately, showed limited efficacy in some studies. For example, the study by Georgina et al. found that in patients with pembrolizumab-treated melanoma, it was ineffective when used alongside a placebo (Ref. 10). Additionally, epacadostat’s in vivo metabolism via the UGT1A9 enzyme results in a short half-life of 2.5 hours, poor hydrophobicity (CLogP = 0.09) and low oral bioavailability (Ref. 57). IDO1 is considered a ‘moonlighting protein’ (Ref. 58), meaning it performs additional functions beyond its catalytic role. Moonlighting proteins can shift between functions by changing their structural conformation in response to various factors, such as changes in redox status, temperature, post-translational modifications (e.g., phosphorylation), cellular localization and interactions with other peptides. Current research suggests that epacadostat can inhibit both enzymatic and non-enzymatic activities of IDO1 (Ref. 59). However, recent findings indicate that epacadostat may inhibit IDO1’s enzymatic activity while stabilizing its lipid form, which promotes tyrosine phosphorylation and binding to the phosphatase SHP-2, potentially contributing to the oncogenic phenotype in SKOV-3 cells (Ref. 60). This suggests that the development of IDO1 inhibitors should target both enzymatic and non-enzymatic activities. Typical TDO2 inhibitors include indoles (e.g., LM10), naphthalenetriazodiones, aminoisoxazoles and other compounds (e.g., catechol, L-adrenaline and p-benzoquinone). However, aminoisoxazole TDO2 inhibitors are less stable in whole blood. Dual IDO1/TDO2 inhibitors, such as indoles, quinone derivatives and indazoles, are also under investigation (Ref. 50).

While traditional drugs have shown some success in clinical trials, they have gradually revealed issues such as adverse reactions and low bioavailability. As a result, researchers have turned to new technologies to address these limitations and enhance the efficacy of traditional treatments.

#### Novel delivery and drug carrier methods combined with traditional inhibitors

To address the shortcomings of traditional inhibitors – such as short half-life, poor hydrophilicity and low cellular uptake – researchers are exploring innovative strategies, including loading effective inhibitors onto novel nanocarriers and delivery systems to enhance drug efficacy. One approach involves the chemical crosslinking of engineered bacteriophage hydrogels (M13 gel) to generate photothermal palladium nanoparticles (PdNPs) in situ on the pVIII coat protein, resulting in M13@Pd gel. Loading the IDO1 inhibitor NLG919 onto this biologically active gel system has shown promise in reversing immune suppression and significantly improving anti-breast cancer chemotherapy outcomes (Ref. 61). Additionally, sonodynamic therapy (SDT)-triggered prodrug-loaded hydrogel delivery systems have been developed to load NLG919. Upon SDT irradiation, the generated singlet oxygen (^1^O_2_) not only induces immunogenic cell death but also disrupts the ^1^O_2_-cleavable linker, activating the NLG919 prodrug precisely (Ref. 62). NLG919 has also been loaded onto other nanozyme therapeutic agents to enhance therapeutic efficacy (Refs 63, 64, 65), and its combination with platinum-based drugs has been explored as a new strategy for combined chemotherapy and immunotherapy in osteosarcoma (Ref. 66). Beyond NLG919, other IDO1 inhibitors have been designed for nanoparticle delivery, improving bioavailability and significantly enhancing immune cascade reactions and tumour microenvironment (TME) remodeling in vivo, resulting in impressive tumour suppression and prolonged survival (Refs 67, 68). Indeed, novel delivery and drug carrier systems have effectively addressed the shortcomings of traditional inhibitors. However, certain design aspects of traditional inhibitors, such as non-enzymatic activity, cannot be simply compensated for by improving delivery methods alone. Further improvements are required in the structural design, starting from the mechanism.

### Targeted inhibition of other enzymes and metabolites in tryptophan metabolism

#### Other enzymes in kynurenine pathway

IDO2 is a 45 kDa enzyme, which has a bis (His) six coordinate heme iron site with two distinct Fe–NHis distances (Ref. 69). It has a large predicted domain with thirteen α-helices and two 3_10_-helices. Mouse IDO2 has a smaller predicted domain with six α-helices, two short β-sheets and three 3_10_-helices (Figure 2C) (Ref. 70). The amino acid similarity between human and mouse IDO1 and IDO2 proteins stands at 43%. Unlike IDO1, IDO2 exhibits little or no tryptophanolytic activity and its immunomodulatory roles in cancer and autoimmune diseases are context-dependent (Ref. 71). Recent studies have suggested that IDO2 may modulate disease processes by virtue of its non-enzymatic activity (Ref. 72). IDO2 is primarily expressed in the liver, kidney, brain, placenta and colon (Figure 3C) (Ref. 73).

Human Interleukin-4-Induced-1 (hIL4I1) is an N-glycosylated secretory protein consisting of 567 amino acids. It is predominantly expressed in immune organs, with the highest levels found in lymph nodes and the spleen. In addition to B cells, hIL4I1 has also been detected in germinal center macrophages, myeloid-derived suppressor cells (MDSC) and antigen-presenting cells (Refs 74, 75). Nevertheless, there remains a scarcity of information regarding the present enzymology and functionality of IL4i1 (Ref. 76), which complicates the design of effective IL4I1 inhibitors.

KMO is a class A FAD monooxygenase typically characterized by a single gene encoding a FAD-binding domain (Ref. 77). hKMO consists of 486 amino acids, has a molecular weight of about 50 kDa, and features two structural domains. In addition to the large FAD-binding region, hKMO also contains a small N-terminal domain, which is composed of an α-helix and an antiparallel β-sheet (Ref. 78).

#### Other enzymes inhibitors

##### IDO2 inhibition

Given that a variety of KP metabolites can affect tumours and that IDO2 has been shown to play an important role in certain cancers (Ref. 71), its potential as a therapeutic target should not be overlooked. IDO2, which is structurally similar to IDO1, has been knocked down in several animal models, leading to tumour growth inhibition, further suggesting its potential for cancer therapy (Ref. 79). As a result, increasing attention is being placed on the development of combined inhibitors targeting both IDO1 and IDO2. The dual inhibitor of IDO1/IDO2, IDO1/IDO2-IN-1, significantly inhibits tumour progression. Notably, in a xenograft mouse model, IDO1/IDO2-IN-1’s in vivo antitumour potency (tumour growth inhibition [TGI] TGI = 69.7%) was much greater than epacadostat’s (TGI = 49.4%), highlighting the benefits of dual IDO1/IDO2 inhibitors in tumour immunotherapy (Ref. 80). We are optimistic about the future clinical applications of this dual inhibitor. However, since IDO2 is downregulated in some tumours (e.g., cervical cancer (Ref. 81)), we propose that the development of selective IDO2 inhibitors should carefully distinguish between IDO1 and IDO2 to enable more personalized therapy.

##### IL4i1 inhibition

In many tumours, IDO1 is highly expressed, AhR levels are also elevated. Sadik et al. analyzed 32 types of tumours using weighted gene co-expression network analysis (WGCNA) and found that in 9 of them, other tryptophan metabolism enzymes activated the aryl hydrocarbon receptor, specifically IL4i1 (Ref. 82). Thus far, tryptophan metabolism enzyme inhibitors have not been successful in clinical trials, possibly due to the overlooked role of IL4i1. In the tumour microenvironment, especially in MDSC, IL4i1 and IDO1 show overlapping expression patterns. We speculate that blocking IDO1 is still a viable supplementary treatment. Consideration must be given to the overlapping effects of IL4i1, as simultaneous inhibition of both enzymes may be necessary for a positive impact in cancer treatment (Ref. 83). Several small molecule inhibitors are being designed (Ref. 84), but given the limited understanding of IL4i1’s role in regulating tumour immunity, we believe further basic research is essential to better inform drug development.

##### Other enzymes inhibition and metabolites degradation products

Utilizing a pharmacologically optimized enzyme, polyethylene glycolylated kynureninase (PEG-KYNase), which degrades kynurenine (Kyn) into non-toxic and easily excreted metabolites, has shown promise in preventing tumour development. Combining PEG-KYNase with approved checkpoint inhibitors or cancer vaccines has demonstrated notable therapeutic efficacy in treating large B16-F10 melanoma, 4T1 breast cancer and CT26 colon cancer tumours (Ref. 85). Due to restricted bioavailability and the inherent instability of proteins in vivo, maintaining sufficiently high concentrations of KYNase in TME has been challenging. Some studies have addressed this issue by loading KYNase onto biodegradable and implantable nanoparticle carriers called ‘BIND,’ enabling sustained release around the tumour (Ref. 86). A different immunomodulatory pathway linked to decreased KMO expression and increased KYNA synthesis, in addition to IDO1, also leads to defective effector CD4^+^ T-cell responses. This indicates that KMO could be a viable target for cancer treatment (Ref. 87). Research by Kesarwani et al. found that tumours in Kynu^−/−^ glioma mice exhibited higher levels of CD8^+^ CD69^+^ T-cell infiltration and significantly reduced expression of CD206^+^ M2 macrophages. This might be explained by the transcription factor Foxo1 being phosphorylated and degraded by QA. Foxo1 binds to the promoter of *PPARγ* and inhibits its production endogenously. Therefore, they propose that targeting downstream tryptophan metabolism may alter the immune characteristics of tumours more effectively than solely targeting IDO1 or TDO2 (Ref. 88).

### Inhibitors derived from mechanisms regulating tryptophan metabolism to modulate the microenvironmen

Metabolic changes in the tumour microenvironment not only affect the biological activity of tumour cells, making them more aggressive in terms of migration and proliferation, but also influence the immune response. These changes can either inhibit tumour development or support tumour immune escape (Ref. 89). A better knowledge of the variables affecting the local immunological balance within TME will be necessary to enhance clinical responses to immune checkpoint inhibition. Stewart et al. integrated multi-omics data such as single-cell RNA sequencing and spatial transcriptomics of classical Hodgkin’s lymphoma, revealing the an immune microenvironment rich in classical monocytes, macrophages and DCs infiltration. Among them, conventional dendritic cells (cDCs) and monocytes express immune checkpoint PD-L1, TIM-3 and IDO (Ref. 90). This suggests that tryptophan metabolism not only affects tumour cells but also regulates the immune microenvironment through immune cell expression. Meanwhile, some scholars characterized the tumour microenvironment where cancer cells overexpressing the IDO1 gene are located through matrix-assisted laser desorption/ionization mass spectrometry imaging. The findings indicate that Trp depletion and Kyn elevation suppress T-cell effector function and metabolism, while fostering a regulatory T-cell phenotype, M2 macrophages and the generation of tolerant dendritic cells (Ref. 91).

#### T cells

γδT cells are immune cells that recognize cancer antigens and belong to the atypical T-cell family. Unlike conventional T cells, their ability to recognize antigens is not limited by major histocompatibility complex (MHC) I and II, and they exhibit potent cytotoxic effects against cancer cells, tumour stem cells, and solid tumours while protecting normal tissues (Ref. 92). Kyn inhibits degranulation and cytotoxicity of γδT cells of pancreatic ductal adenocarcinoma (Ref. 93). FOXP3^+^ Tregs is a distinct subpopulation of lymphocytes that promote tumourigenesis by clearing self-reactive T cells from the thymus and peripheral organs. FOXP3 loss-of-function mutations in both animal models and humans result in the inability to differentiate into Tregs, leading to highly aggressive, lethal, systemic immune-mediated inflammatory disease. Kyn reduces Th1/Th22 development while massively inducing AhR-dependent cells to produce FoxP3^+^ Tregs, and IDO inhibitors reverse that process (Refs 94, 95, 96, 97, 98). The presence of KynA also suppresses Th17 cells’ expression of IL-23 and IL-17 (Ref. 99). Inhibiting KMO increases CD4^+^ T-cell counts in SIV-infected rhesus monkeys, suggesting that KP metabolism regulates CD4^+^ T cells (Ref. 100). Additionally, KynA strongly inhibits CD4^+^ T-cell proliferation and IFN-γ release in melanoma (Ref. 87). Other metabolites like L-Kyn, 3-HK, 3HAA and QA also can inhibit T-cell activation and proliferation (Refs 101, 102, 103). Nevertheless, it has been proposed that kynurenine uses SLC7A5 to pass through the T-cell membrane. Consequently, only T cells that express SLC7A5 may be affected by Kyn (Ref. 104). Trp catabolic enzymes also play a role in driving adaptive immune resistance mechanisms by inhibiting the cytolytic function of CD8^+^ T cells (Refs 105, 106). According to a study, Kyn-AhR increases the expression of the programmed cell death protein 1 (PD-1) on CD8^+^ T lymphocytes, indicating a possible therapeutic approach to target this network in cancer (Refs 107, 108).

#### Tumour-associated macrophages (TAMs)

TAMs are the most prevalent immune cells in the majority of tumours and make up the diverse and changeable cell type of TME, making up around 30% of all the cells in tumour tissues (Ref. 109). TAMs give malignant cells nutritional assistance, which promotes disease development and treatment resistance (Ref. 110). TDO2 and IDO1 silencing in mouse glioma cells decreased the expression of the immunosuppressive gene *Arg-1* (M2 polarization) in TAM, suggesting that Kyn secreted by gliomas may regulate the phenotype of TAMs (Ref. 111). Low systemic kynurenine levels are linked to a lower overall survival rate. Glioblastoma cell-produced Kyn activates AhR in TAMs to enhance *CCR2* expression, drives TAM recruitment in response to CCL2, drives *KLF4* expression, and inhibits NF-κB expression in TAMs (Refs 111, 112). IDO1 expression and Kyn metabolism may promote autophagy in cervical cancer cells and facilitate its clearance by macrophages (Ref. 113).

#### B cells

The direct impact of Tryptophan metabolism on B-cell development and proliferation remains unclear. Some theories suggest that IDO1 plays a crucial role as a feedback mechanism that limits antibody production and B-cell proliferation. Additionally, IDO1 is thought to promote apoptosis and negatively regulate B cell proliferation in response to LPS stimulation (Ref. 114). MDSC expressing IDO have been shown to promote B-cell proliferation (Ref. 115). Recently, it has been demonstrated that IDO1 and IDO2 have opposing functions in the regulation of B cells, with IDO2 enhancing inflammatory B-cell responses and IDO1 suppressing them (Ref. 116).

#### Natural killer cells (NK cells)

L-Kyn inhibits the cytokine-mediated upregulation of specific trigger receptors, such as NKp46 and NKG2D, which are essential for NK cells to identify and kill target cells, thereby facilitating tumour immune escape (Refs 117, 118). In multiple myeloma, KMO impairs the activation of defective plasmacytoid dendritic cells and stimulates the cytolytic activity of certain NK cells and cytotoxic T lymphocytes against tumour cells (Ref. 119). Additionally, altered 3-HAA concentrations promote the activation of T and NK cells by increasing the expression of *CXCL11* and KLRD1 (Ref. 120). IDO in thyroid cancer cells produces Kyn, which leads to NK cells dysfunction by a possible mechanism that reduces NK cells function through the signal transduction and transcriptional activator *STAT1* and *STAT3* pathways (Ref. 121). Furthermore, the AhR-IDO axis, modulated by both acute and long-term endurance exercise, plays a role in controlling NK cell activity and contributing to immunological modulation (Ref. 122).

#### Dendritic cells

Binding of IFN-γ to IDO promoter region induces IDO expression in DCs (Refs 123, 124). DC regulation of T cells induction may be through intracellular IDO, and the data suggest that under specific conditions, cDC1 selectively expresses IDO, which inhibits T cells proliferation and triggers tumour immune escape (Ref. 125). Kyn-AhR helps maintain the DC tolerance phenotype by maintaining self-amplification of IDO (Ref. 126). Additionally, downregulation of IDO in DCs leads to increased CD4^+^ T-cell proliferation and a reduction in Treg cells (Ref. 127). KynA plays an crucial role in regulating DC function by blocking *GPCR35* and the downregulation of IFN-γ and cAMP signaling (Ref. 99).

#### Cancer associated fibroblasts (CAFs)

By promoting the growth, invasion and metastasis of cancer cells, CAFs contribute significantly to the advancement of tumours (Refs 128, 129). Itoh et al. found that CAFs supernatants stimulate normal fibroblasts to become CAF-educated fibroblasts, which further express IDO1 and KYNU by secreting extracellular matrix proteins, ultimately leading to tumour cell dissemination (Ref. 130). CAFs up-regulate tryptophan *TDO2* expression, leading to enhanced secretion of Kyn. Kyn produced by CAFs can upregulate *AhR* expression and activate AhR-AKT-STAT3, which causes tumour cell proliferation (Ref. 131). PDPN^+^ CAFs (podoplanin-positive CAFs) promote resistance of *HER2*-positive breast cancer to trastuzumab by secreting immunosuppressive factors IDO1 and TDO2 (Ref. 132).

#### Local or global Tumourtumour microenvironment

The regulatory role of tryptophan metabolism on specific cells within the microenvironment appears relatively clear in current research (Ref. 133). However, existing studies may have overlooked some factors that should be taken into account. For example, inhibition of T-cell proliferation requires a local microenvironment with a low tryptophan content, whereas human plasma concentrations vary from 50 to 100 μM, so how are localized low concentrations of tryptophan generated? (Refs 134, 135). This discrepancy suggests that future studies must consider the tumour microenvironment as a whole (Refs 133, 134, 135).

As research progressed, studies began to view the local balance of the tumour microenvironment as a whole, for instance, considering antigen-presenting cells collectively. When tryptophan metabolism enzymes are active, antigen-presenting cells that would normally produce inflammatory cytokines (such as IL-12) instead generate inhibitory cytokines (such as IL-10). This suggests that the upregulation of tryptophan metabolism enzymes can alter the characteristics of antigen-presenting cells and shift the entire local environment from immunogenicity to tolerance (Ref. 136). The communication between immune cells has also gained more attention. Luis et al. established that the contact between Tregs and tumour-associated macrophages is necessary for the immunological suppression mediated by IDO-Kyn-AhR (Ref. 137). Mature DCs can express IDO1 and interact with tumour-reactive exhausted CD8^+^ T- cells and Tregs, collectively forming a malignant immune suppression cycle and mediating immune escape in cervical cancer (Ref. 138). These findings suggest that tryptophan metabolism does not act independently on specific immune cells but instead facilitates communication among various cell types, creating an environment conducive to tumour immune evasion. Various alterations in tumour cells and the surrounding tumour microenvironment arise from abnormalities in Tryptophan metabolism in gliomas. Glioblastomas may be able to evade immune system responses due to these metabolic alterations, thereby promoting tumour growth (Ref. 139). To acquire a better insight into the global influence of tryptophan metabolism on the microenvironment, Zhang et al (Ref. 140) Analyzed data from multiple public databases and 1,523 patient samples. The results suggest that the high-scoring group of tryptophan metabolism-related genes is correlated with increased infiltration of immune cells and a “hot’‘ immune phenotype, which is associated with shorter overall survival in this group. This indicates that Trp and its metabolism play an important role in reshaping the immune landscape. Similarly, research in low-grade gliomas has also demonstrated this phenomenon (Ref. 141). The above results collectively indicate that future research on tryptophan metabolism is transitioning from its inhibitory effects on specific microenvironment cells to interactions among multiple cells and even the microenvironment as a whole (Refs 136, 137, 138, 139, 140, 141).

**Figure 4.**
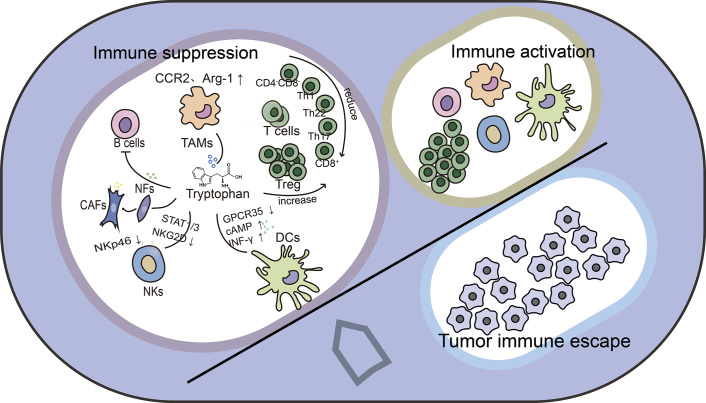
Effect of tryptophan metabolism in the tumor microenvironment. Multiple metabolites produced in tryptophan metabolism, particularly in the kynurenine pathway, act on B cells, T cells, tumor-associated macrophages, fibroblasts, and NK cells to transform the immune activation state into an immunosuppressive microenvironment, thereby facilitating immune escape from the tumour.

In conclusion, the metabolism of tryptophan is crucial for controlling the TME (Refs 136, 137, 138, 139, 140, 141). Its regulatory role in tumours extends beyond the tumour cells themselves and is also important for other cells in the TME (Figure 4). A comprehensive understanding of the regulatory role and mechanisms of tryptophan metabolism in the complex microenvironment is essential for developing more effective therapeutic strategies (Ref. 142).

#### The potential effects of bacterial tryptophan metabolism on tumours

The heterogeneity of bacterial complement in cancer patients may influence the response to immunomodulators (Ref. 143). The impact of intratumoural bacteria on tumours is gradually gaining attention. Indole, produced through bacterial degradation of dietary tryptophan by tryptophanase or phenyllacetate dehydratase-mediated breakdown of phenylalanine, could involve other as-yet undiscovered pathways. While this paper focuses on KP metabolites, products of Tryptophan metabolism have been shown to activate immune regulation through receptors similar to those in humans. For instance, indole-3-aldehyde (I3A), a dietary tryptophan metabolite released by *Lactobacillus reuteri*, induces CREB activity, promoting anti-tumour immunity (Ref. 144). Furthermore, researchers have found that the activation of macrophage AhR activity depends on *lactobacilli* metabolizing dietary Trp into indole. This can reduce the growth of pancreatic ductal adenocarcinoma, enhance the efficacy of immune checkpoint blockade, and increase the frequency of IFN-γ^+^ CD8^+^ T cells within tumours – without requiring macrophage-specific Tryptophan metabolism (Ref. 145). This raises questions about the source of tryptophan metabolites that influence the tumour microenvironment. Additionally, the discovery that *Salmonella* inhibits *IDO* expression has led researchers to explore whether it is possible to use microbiota to modulate the tumour immune microenvironment (Ref. 146). At the same time, interference with *IDO1* affects *Staphylococcus aureus* replication (Ref. 147), prompting further questions on whether inhibiting tryptophan metabolism in bacteria might counteract the beneficial effects of intratumoural bacteria for tumours. These considerations will be crucial when designing future inhibitors.

#### AhR inhibition

Although there are still questions about IDO1 inhibition, there is a lot of preclinical data supporting the ongoing development of inhibitors targeting the Trp-Kyn-AhR pathway to enhance immune checkpoint blockade and other cancer treatments (Ref. 148). A highly selective exogenous or endogenous AhR ligand inhibitor, BAY 2416964, exhibits good tolerability upon oral administration in vivo, inducing a pro-inflammatory tumour microenvironment and demonstrating anti-tumour efficacy in syngeneic mouse models (Ref. 149). Additionally, activation of AhR by tryptophan metabolites can induce T-cell expression of PD1, an effect significantly abolished by the AhR antagonist CH223191. The role of KP metabolites and key enzymes in the tumour immune-suppressive microenvironment is primarily mediated through activation of the AhR receptor, which was one potential limitation of past IDO1 inhibitor clinical trials – some inhibitors themselves act as AhR agonists (Ref. 150). Hence, future drug design should aim to avoid this issue. However, AhR inhibitors also remain controversial, primarily due to the dual nature of AhR. Besides serving as a ligand-activated transcription factor that promotes tumour immune evasion, AhR can also act as a ligand-dependent E3 ubiquitin ligase, promoting the degradation of β-catenin by tryptophan metabolites in intestinal carcinogenesis, thereby inhibiting cancer development (Ref. 151). Therefore, optimizing the anticancer properties of AhR will be a key focus in future research.

#### Compensatory metabolic alterations interference

In addition to the paradoxical role of AhR in regulating tryptophan metabolism, the immunosuppressive environment induced by aberrant activation of the KP can be counterbalanced by compensatory metabolic changes triggered by KP activation (Ref. 152). Inhibition of IDO1 effectively blocks tryptophan degradation through the kynurenine pathway, but this also leads to metabolic adaptation, redirecting tryptophan catabolism toward the serotonin pathway (Ref. 153). This contributes to the clinical failure of IDO1 inhibitors by raising nicotinamide adenine dinucleotide levels, which in turn impair T-cell proliferation and function (Ref. 153). Combining IDO1 inhibition with A2a/A2b receptor blockade enhances the survival of ovarian cancer mice overexpressing IDO1 and strengthens anti-tumour immune features, suggesting the importance of understanding the regulatory role and mechanisms of tryptophan metabolism in the immune environment for devising new therapeutic strategies (Ref. 153). It is crucial to consider not only the modulation of the tumour microenvironment by the KP itself, but also the potential impact of compensatory metabolic alterations following KP inhibition on the immune landscape.

### Combination therapy approaches with other anticancer drugs

#### Combination with other anticancer therapy

Combining targeted therapies with other drugs often enhances the efficacy of treatments aimed at modulating tryptophan metabolism. Song et al. developed a self-amplifying, ROS-responsive nanocarrier co-loaded with the immunogenic inducer paclitaxel and the IDO1 inhibitor 1-MT. This nano-platform demonstrated efficient immunogenic cell death, promoting robust T-cell infiltration and triggering anti-tumour immune responses. In 4T1 tumour-bearing mice, IDO inhibition reduced the immune-suppressive tumour microenvironment by attenuating Treg and M2-TAM infiltration, resulting in substantial primary tumour regression and reduced lung metastasis. These results imply that employing ROS-amplifying nanoplatforms to co-deliver immunogenic inducers and IDO inhibitors holds significant promise for advancing tumour chemotherapeutic immunotherapy (Ref. 154).

Numerous cancer types can go into remission as a result of viral infections that occur naturally in humans. Clinical trials for the oncolytic virus Delta-24-RGD are presently underway to treat liver metastases (NCT04714983) and malignant gliomas (NCT03714334). When combined with IDO inhibitors, this approach enhances human glioblastoma’s resistance to oncolytic viruses, thereby improving the efficacy of immunotherapy. This suggests that IDO1 inhibition’s molecular and immunological effects could enhance the results of other virotherapy-treated malignancies (Ref. 155).

One of the most well-known combination therapies is the pairing of epacadostat and pembrolizumab. While this combination demonstrates good tolerability, its anti-tumour activity has been limited in clinical trials. Phase III clinical trial of IDO1 inhibitor epacadostat in combination with PD-1 checkpoint inhibitor pembrolizumab for melanoma declared a failure in 2018 (Ref. 10). Relevant data suggest that achieving maximal suppression of IDO1 activity in the context of anti-PD-1 therapy may require higher doses of epacadostat than those used in previous studies (Ref. 156). Increasing the local concentration of IDO1 inhibitors is indeed effective, and understanding the mechanism behind the need for higher concentrations of IDO1 inhibitors in anti-PD-1 therapy can better optimize treatment regimens. Another Phase III trial (KEYNOTE-669/ECHO-304) of pembrolizumab in combination with epacadostat for recurrent/metastatic squamous cell carcinoma of the head and neck found that the combination had a safety profile comparable to pembrolizumab monotherapy. While epacadostat reduced the elevated kynurenine levels associated with pembrolizumab treatment, it did not lower them to the levels seen in healthy volunteers. The authors hypothesized that doses of epacadostat ≥600 mg twice daily may be required to counteract the anti-PD-1-induced upregulation of IFN-γ-generated IDO1 (Ref. 157).

Recent research has revealed that the KP interacts with and modulates the activity of several other signaling pathways. Pharmacologically targeting the KP can indirectly influence anticancer defense mechanisms, as well as impact inflammatory responses and tumour progression (Ref. 7). A promising strategy could involve combining IDO inhibitors with drugs that block other signaling pathways, such as those associated with PIK3CA mutations, which often occur alongside IDO1 overexpression (Ref. 158).

#### Chimeric antigen receptor T-cell therapy (CAR-T)

Several challenges have impeded the use of CAR-T treatment for solid malignancies. However, combining IDO1 inhibition with immune checkpoint blockade has shown promising results in both preclinical and clinical studies, offering long-lasting therapeutic benefits. For instance, in colorectal cancer mouse models, miR-153 inhibits IDO1 expression in tumour cells, thereby enhancing the efficacy of CAR-T therapy (Ref. 159). IDO1 suppresses the function of anti-GD2 CAR T cells and NK cells, primarily by preventing their production of IFN-γ. Combining NK or GD2.CAR T cells with IDO1 inhibitors provide a fresh approach to immunotherapy’s long-term efficacy (Ref. 160).

#### Immune-modulating vaccines

One promising strategy to cancer immunotherapy is antigen peptide vaccination. However, the low antigenicity and insufficient immune response stimulation often limit its effectiveness. To overcome these challenges, cationic liposomes co-delivering tumour vaccines and IDO inhibitors have been developed. These liposomes not only stimulate anti-tumour T-cell immunity but also help reverse the immune-suppressive tumour microenvironment, providing a promising platform for cancer immunotherapy (Ref. 161). Targeting both malignant and regulatory cells, the T-win® immune-modulating anti-cancer medicines work by triggering the body’s natural anti-Tregs. Because they can identify proteins like IDO or PD-L1 that are produced by regulatory immune cells, anti-regulatory T cells are naturally occurring T cells that can target these cells directly. In the end, they draw pro-inflammatory cells to the tumour microenvironment, which has a direct effect on immune suppression mechanisms and may change tumour antigen tolerance. IFN-γ causes circulating IDO or PD-L1-specific anti-Tregs to proliferate, and pre-incubation with IFN-γ improves their sensitivity to target cell recognition of IDO or PD-L1-specific anti-Tregs. Thus far, vaccines that target IDO or PD-L1 have been shown to be safe and to have little harm (Ref. 162). A deeper understanding of tryptophan metabolism’s regulatory role in the tumour microenvironment can aid in the development of new immunotherapeutic approaches.

In conclusion, tryptophan metabolism plays a crucial role in the initiation and progression of tumours, intimately intertwined with shaping a microenvironment conducive to tumour evasion. As scholars delve deeper into its mechanisms in tumour regulation, strategies targeting tryptophan metabolism for cancer treatment are becoming increasingly refined.

## Abbreviations

3-HAA: 3-Hydroxyanthranilic acid
3-HK: 3-Hydroxy-kynurenine
5-HT: 5-Hydroxytryptamine
AA: Anthranilic acid
AADC: L-amino acid decarboxylase
AFMID: Arylformamidase
AhR: Aryl hydrocarbon receptor
CAFs: Cancer associated fibroblasts
CAR-T: Chimeric antigen receptor T-cell therapy
DCs: Dendritic cells
DLBCL: Diffuse large B cell lymphoma
FICZ: 6-Formylbenzo[3,2-b]carbazole
HAAO: 3-Hydroxyanthranilate 3,4-dioxygenase
hIL4I1: Human interleukin-4-induced-1
IDO: Indoleamine 2,3 dioxygenase
KA: Kynurenic acid
KATI-KATIII: Kynurenic aminotransferase
KMO: Kynurenine 3-monooxygenase
KP: Kynurenine pathway
KTR: Kyn/Trp ratio
Kyn: Kynurenine
KYNU: Kynureninase
MDSC: Myeloid-derived suppressor cells
MHC: Major histocompatibility complex
NFK: N-formyl kynurenine
NK cells: Natural killer cells
PD: Progressive disease
QA: Quinolinic acid
TAMs: Tumour-associated macrophages
TDO2: Tryptophan 2,3-dioxygenase 2
TGI: Tumour growth inhibition
TME: Tumour microenvironment
TPH: Tryptophan hydroxylase
Trp: Tryptophan
XA: Xanthurenic acid

## References

[1] Bray F, Laversanne M, Sung H, Ferlay J, Siegel RL, Soerjomataram I and Jemal A (2024) Global cancer statistics 2022: GLOBOCAN estimates of incidence and mortality worldwide for 36 cancers in 185 countries[J]. CA: A Cancer Journal for Clinicians 74(3), 229–263. 38572751 10.3322/caac.21834

[2] Wahida A, Buschhorn L, Fröhling S, Jost PJ, Schneeweiss A, Lichter P and Kurzrock R (2023) The coming decade in precision oncology: Six riddles[J]. Nature Reviews Cancer 23(1), 43–54. 36434139 10.1038/s41568-022-00529-3

[3] Ye F, Dewanjee S, Li Y, Jha NK, Chen ZS, Kumar A, Vishakha BT, Jha SK and Tang H (2023) Advancements in clinical aspects of targeted therapy and immunotherapy in breast cancer[J]. Molecular Cancer 22(1), 105. 37415164 10.1186/s12943-023-01805-y PMC10324146

[4] Pavlova NN and Thompson CB (2016) The emerging hallmarks of cancer metabolism[J]. Cell Metabolism 23(1), 27–47. 26771115 10.1016/j.cmet.2015.12.006 PMC4715268

[5] Stine ZE, Schug ZT, Salvino JM and Dang CV (2022) Targeting cancer metabolism in the era of precision oncology[J]. Nature Reviews Drug Discovery 21(2), 141–162. 34862480 10.1038/s41573-021-00339-6 PMC8641543

[6] Xue C, Li G, Zheng Q, Gu X, Shi Q, Su Y, Chu Q, Yuan X, Bao Z, Lu J and Li L (2023) Tryptophan metabolism in health and disease[J]. Cell Metabolism 35(8), 1304–1326. 37352864 10.1016/j.cmet.2023.06.004

[7] Stone TW and Williams RO (2023) Interactions of IDO and the kynurenine pathway with cell transduction systems and metabolism at the inflammation-cancer interface[J]. Cancers 15(11), 2895. 37296860 10.3390/cancers15112895 PMC10251984

[8] Girithar HN, Staats Pires A, Ahn SB, Guillemin GJ, Gluch L and Heng B (2023) Involvement of the kynurenine pathway in breast cancer: Updates on clinical research and trials[J]. British Journal of Cancer 129(2), 185–203. 37041200 10.1038/s41416-023-02245-7 PMC10338682

[9] Zhang Y, Hu Z, Zhang J, Ren C and Wang Y (2022) Dual-target inhibitors of indoleamine 2, 3 dioxygenase 1 (Ido1): A promising direction in cancer immunotherapy[J]. European Journal of Medicinal Chemistry 238, 114524. 35696861 10.1016/j.ejmech.2022.114524

[10] Long GV, Dummer R, Hamid O, Gajewski TF, Caglevic C, Dalle S, Arance A, Carlino MS, Grob J-J, Kim TM, Demidov L, Robert C, Larkin J, Anderson JR, Maleski J, Jones M, Diede SJ and Mitchell TC (2019) Epacadostat plus pembrolizumab versus placebo plus pembrolizumab in patients with unresectable or metastatic melanoma (ECHO-301/KEYNOTE-252): A phase 3, randomised, double-blind study[J]. The Lancet Oncology 20(8), 1083–1097. 31221619 10.1016/S1470-2045(19)30274-8

[11] Badawy AA (2022) Tryptophan metabolism and disposition in cancer biology and immunotherapy[J]. Bioscience Reports 42(11), BSR20221682. 36286592 10.1042/BSR20221682 PMC9653095

[12] Li Y, Hu N, Yang D, Oxenkrug G and Yang Q (2017) Regulating the balance between the kynurenine and serotonin pathways of tryptophan metabolism[J]. FEBS Journal 284(6), 948–966. 28118532 10.1111/febs.14026

[13] Platten M, Nollen EAA, Rohrig UF, Fallarino F and Opitz CA (2019) Tryptophan metabolism as a common therapeutic target in cancer, neurodegeneration and beyond[J]. Nature Reviews Drug Discovery 18(5), 379–401. 30760888 10.1038/s41573-019-0016-5

[14] Stone TW, Stoy N and Darlington LG (2013) An expanding range of targets for kynurenine metabolites of tryptophan[J]. Trends in Pharmacological Sciences 34(2), 136–143. 23123095 10.1016/j.tips.2012.09.006

[15] Maynard A, Mccoach CE, Rotow JK, Harris L, Haderk F, Kerr DL, Yu EA, Schenk EL, Tan W, Zee A, Tan M, Gui P, Lea T, Wu W, Urisman A, Jones K, Sit R, Kolli PK, Seeley E, Gesthalter Y, Le DD, Yamauchi KA, Naeger DM, Bandyopadhyay S, Shah K, Cech L, Thomas NJ, Gupta A, Gonzalez M, Do H, Tan L, Bacaltos B, Gomez-Sjoberg R, Gubens M, Jahan T, Kratz JR, Jablons D, Neff N, Doebele RC, Weissman J, Blakely CM, Darmanis S and Bivona TG (2020) Therapy-induced evolution of human lung cancer revealed by single-cell RNA sequencing[J]. Cell 182(5), 1232, e1222–1251. 10.1016/j.cell.2020.07.017 PMC7484178 32822576

[16] Bishnupuri KS, Alvarado DM, Khouri AN, Shabsovich M, Chen B, Dieckgraefe BK and Ciorba MA (2019) IDO1 and kynurenine pathway metabolites activate PI3K-AKT signaling in the neoplastic colon epithelium to promote cancer cell proliferation and inhibit apoptosis[J]. Cancer Research 79(6), 1138–1150. 30679179 10.1158/0008-5472.CAN-18-0668 PMC6420842

[17] Ma W, Ye L, Zhong C, Li J, Ye F, Lv L, Yu Y, Jiang S and Zhou P (2022) Kynurenine produced by tryptophan 2,3-dioxygenase metabolism promotes glioma progression through an aryl hydrocarbon receptor-dependent signaling pathway[J]. Cell Biology International 46(10), 1577–1587. 35702760 10.1002/cbin.11833

[18] Jin H, Zhang Y, You H, Tao X, Wang C, Jin G, Wang N, Ruan H, Gu D, Huo X, Cong W and Qin W (2015) Prognostic significance of kynurenine 3-monooxygenase and effects on proliferation, migration, and invasion of human hepatocellular carcinoma[J]. Scientific Reports 5, 10466. 26099564 10.1038/srep10466 PMC4479133

[19] Ala M (2021) The footprint of kynurenine pathway in every cancer: A new target for chemotherapy[J]. European Journal of Pharmacology 896, 173921. 33529725 10.1016/j.ejphar.2021.173921

[20] Jin J, Byun JK, Choi YK and Park KG (2023) Targeting glutamine metabolism as a therapeutic strategy for cancer[J]. Experimental and Molecular Medicine 55(4), 706–715. 37009798 10.1038/s12276-023-00971-9 PMC10167356

[21] Lee R, Li J, Li J, Wu CJ, Jiang S, Hsu WH, Chakravarti D, Chen P, Labella KA, Li J, Spring DJ, Zhao D, Wang YA and Depinho RA (2022) Synthetic essentiality of tryptophan 2,3-dioxygenase 2 in APC-mutated colorectal cancer[J]. Cancer Discovery 12(7), 1702–1717. 35537038 10.1158/2159-8290.CD-21-0680 PMC9262860

[22] Du L, Xing Z, Tao B, Li T, Yang , Li W, Zheng Y, Kuang C and Yang Q (2020) Both IDO1 and TDO contribute to the malignancy of gliomas via the Kyn-AhR-AQP4 signaling pathway[J]. Signal Transduction and Targeted Therapy 5(1), 10. 32296044 10.1038/s41392-019-0103-4 PMC7033114

[23] Walczak K, Langner E, Makuch-Kocka A, Szelest M, Szalast K, Marciniak S and Plech T (2020) Effect of tryptophan-derived ahr ligands, kynurenine, kynurenic acid and ficz, on proliferation, cell cycle regulation and cell death of melanoma cells-in vitro studies[J]. International Journal of Molecular Sciences 21(21), 7946. 33114713 10.3390/ijms21217946 PMC7663343

[24] Lai MH, Liao CH, Tsai NM, Chang KF, Liu CC, Chiu YH and Huang KC (2021) Surface expression of kynurenine 3-monooxygenase promotes proliferation and metastasis in triple-negative breast cancers[J]. Cancer Control 28, 10732748211009245. 33887987 10.1177/10732748211009245 PMC8204454

[25] Yu C, Rao D, Zhu H, Liu Q, Huang W, Zhang L, Liang H, Song J and Ding Z (2021) Tdo2 was downregulated in hepatocellular carcinoma and inhibited cell proliferation by upregulating the expression of p21 and p27[J]. BioMed Research International 2021, 4708439. 34423034 10.1155/2021/4708439 PMC8378971

[26] Oweira H, Lahdou I, Mehrle S, Khajeh E, Nikbakhsh R, Ghamarnejad O, Terness P, Reissfelder C, Sadeghi M and Ramouz A (2022) Kynurenine is the main metabolite of tryptophan degradation by tryptophan 2,3-dioxygenase in hepg2 tumor cells[J]. Journal of Clinical Medicine 11(16), 4794. 36013032 10.3390/jcm11164794 PMC9410271

[27] Miyazaki T, Chung S, Sakai H, Ohata H, Obata Y, Shiokawa D, Mizoguchi Y, Kubo T, Ichikawa H, Taniguchi H, Aoki K, Soga T, Nakagama H and Okamoto K (2021) Stemness and immune evasion conferred by TDO2-AHR pathway are associated with liver metastasis of colon cancer[J]. Cancer Science 13(1), 170–181. 10.1111/cas.15182 PMC8748246 34714577

[28] Low HY, Lee YC, Lee YJ, Wang HL, Chen YI, Chien PJ and Li ST (2020) Reciprocal regulation between indoleamine 2,3-dioxigenase 1 and notch1 involved in radiation response of cervical cancer stem cells[J]. Cancers 12(6), 1547. 32545442 10.3390/cancers12061547 PMC7352771

[29] Xiong J, Zhang X, Zhang Y, Wu B, Fang L, Wang N, Yi H, Chang N, Chen L and Zhang J (2020) Aryl hydrocarbon receptor mediates JAK2/STAT3 signaling for non-small cell lung cancer stem cell maintenance[J]. Experimental Cell Research 396(1), 112288. 32941808 10.1016/j.yexcr.2020.112288

[30] Liang F, Wang G-Z, Wang Y, Yang Y-N, Wen Z-S, Chen D-N, Fang W-F, Zhang B, Yang L, Zhang C, Han S-C, Yang F-Y, Wang D, Liang L-J, Wang Z, Zhao Y, Wang C-L, Zhang L and Zhou G-B (2022) Tobacco carcinogen induces tryptophan metabolism and immune suppression via induction of indoleamine 2,3-dioxygenase 1[J]. Signal Transduction and Targeted Therapy 7(1), 311. 36068203 10.1038/s41392-022-01127-3 PMC9448807

[31] Wang L, Tang W, Yang S, He P, Wang J, Gaedcke J, Strobel , Azizian A, Ried T, Gaida MM, Yfantis HG, Lee DH, Lal A, Van Den Eynde BJ, Alexander HR, Ghadimi BM, Hanna N and Hussain SP (2020) NO^`^ /RUNX3/kynurenine metabolic signaling enhances disease aggressiveness in pancreatic cancer[J]. International Journal of Cancer 146(11), 3160–3169. 31609478 10.1002/ijc.32733 PMC8189162

[32] Morita N, Hoshi M, Hara T, Ninomiya S, Enoki T, Yoneda M, Tsurumi H and Saito K (2021) Viability of diffuse large B-cell lymphoma cells is regulated by kynurenine 3-monooxygenase activity[J]. Oncology Letters 22(5), 790. 34584567 10.3892/ol.2021.13051 PMC8461759

[33] Zhang D, Ning J, Ramprasath T, Yu C, Zheng X, Song P, Xie Z and Zou MH (2022) Kynurenine promotes neonatal heart regeneration by stimulating cardiomyocyte proliferation and cardiac angiogenesis[J]. Nature Communications 13(1), 6371. 10.1038/s41467-022-33734-7 PMC9606021 36289221

[34] Mondanelli G, Volpi C and Orabona C (2022) Decoding the complex crossroad of tryptophan metabolic pathways[J]. International Journal of Molecular Sciences 23(2), 787–792. 35054973 10.3390/ijms23020787 PMC8776215

[35] Botticelli A, Mezi S, Pomati G, Cerbelli B, Cerbelli E, Roberto M, Giusti R, Cortellini A, Lionetto L, Scagnoli S, Zizzari IG, Nuti M, Simmaco M and Marchetti P (2020) Tryptophan catabolism as immune mechanism of primary resistance to anti-PD-1[J]. Frontiers in Immunology 11, 1243. 32733441 10.3389/fimmu.2020.01243 PMC7358280

[36] Mandarano M, Orecchini E, Bellezza G, Vannucci J, Ludovini V, Baglivo S, Tofanetti FR, Chiari R, Loreti E, Puma F, Sidoni A and Belladonna ML (2021) Kynurenine/tryptophan ratio as a potential blood-based biomarker in non-small cell lung cancer[J]. International Journal of Molecular Sciences 22(9), 4403. 33922388 10.3390/ijms22094403 PMC8122814

[37] Takada K, Kohashi K, Shimokawa M, Haro A, Osoegawa A, Tagawa T, Seto T, Oda Y and Maehara Y (2019) Co-expression of IDO1 and PD-L1 in lung squamous cell carcinoma: Potential targets of novel combination therapy[J]. Lung Cancer 128, 26–32. 30642449 10.1016/j.lungcan.2018.12.008

[38] Moon PK, Tran S and Minhas PS (2019) Revisiting IDO and its value as a predictive marker for anti-PD-1 resistance[J]. Journal of Translational Medicine 17(1), 31. 30658666 10.1186/s12967-019-1784-8 PMC6339344

[39] Meireson A, Ferdinande L, Haspeslagh M, Hennart B, Allorge D, Ost P, Sundahl N, Spaas M, Demeyer A and Brochez L (2021) Clinical relevance of serum Kyn/Trp ratio and basal and IFNγ-upregulated IDO1 expression in peripheral monocytes in early stage melanoma[J]. Frontiers in Immunology 12, 736498. 34557196 10.3389/fimmu.2021.736498 PMC8453201

[40] Lucarelli G, Rutigliano M, Ferro M, Giglio A, Intini A, Triggiano F, Palazzo S, Gigante M, Castellano G, Ranieri E, Buonerba C, Terracciano D, Sanguedolce F, Napoli A, Maiorano E, Morelli F, Ditonno P and Battaglia M (2017, 461) Activation of the kynurenine pathway predicts poor outcome in patients with clear cell renal cell carcinoma[J]. Urologic Oncology 35(7), e415, e427–e461. 10.1016/j.urolonc.2017.02.011 28359744

[41] Song X, Si Q, Qi R, Liu W, Li M, Guo M, Wei L and Yao Z (2021) Indoleamine 2,3-dioxygenase 1: A promising therapeutic target in malignant tumor[J]. Frontiers in Immunology 12, 800630. 35003126 10.3389/fimmu.2021.800630 PMC8733291

[42] Fiore A and Murray PJ (2021) Tryptophan and indole metabolism in immune regulation[J]. Current Opinion in Immunology 70, 7–14. 33418116 10.1016/j.coi.2020.12.001

[43] Song P, Ramprasath T, Wang H and Zou MH (2017) Abnormal kynurenine pathway of tryptophan catabolism in cardiovascular diseases[J]. Cellular and Molecular Life Sciences 74(16), 2899–2916. 28314892 10.1007/s00018-017-2504-2 PMC5501999

[44] Zhai L, Ladomersky E, Lenzen A, Nguyen B, Patel R, Lauing KL, Wu M and Wainwright DA (2018) IDO1 in cancer: A gemini of immune checkpoints[J]. Cellular & Molecular Immunology 15(5), 447–457. 29375124 10.1038/cmi.2017.143 PMC6068130

[45] Luo S, Xu K, Xiang S, Chen J, Chen C, Guo C, Tong Y and Tong L (2018) High-resolution structures of inhibitor complexes of human indoleamine 2,3-dioxygenase 1 in a new crystal form[J]. Acta Crystallographica Section F-Structural Biology Communications 74(Pt 11), 717–724. 10.1107/S2053230X18012955 PMC6213978 30387777

[46] Weng T, Qiu X, Wang J, Li Z and Bian J (2018) Recent discovery of indoleamine-2,3-dioxygenase 1 inhibitors targeting cancer immunotherapy[J]. European Journal of Medicinal Chemistry 143, 656–669. 29220788 10.1016/j.ejmech.2017.11.088

[47] Hornyak L, Dobos N, Koncz G, Karanyi Z, Pall D, Szabo Z, Halmos G and Szekvolgyi L (2018) The role of indoleamine-2,3-dioxygenase in cancer development, diagnostics, and therapy[J]. Frontiers in Immunology 9, 151. 29445380 10.3389/fimmu.2018.00151 PMC5797779

[48] Yang R, Gao N, Chang Q, Meng X and Wang W (2019) The role of IDO, IL-10, and TGF-beta in the HCV-associated chronic hepatitis, liver cirrhosis, and hepatocellular carcinoma[J]. Journal of Medical Virology 91(2), 265–271. 29611873 10.1002/jmv.25083

[49] Van Baren N, Van Den Eynde BJ. Tryptophan-degrading enzymes in tumoral immune resistance[J]. Frontiers in Immunology,2015, 6: 34. 25691885 10.3389/fimmu.2015.00034 PMC4315104

[50] 俊敏 董 and 站柱 刘 (2020) 基于靶标 IDO1/TDO 的抑制剂研究进展. 药学学报, 1265–1278.

[51] Leeds JM, PJB , McGeehan GM, Brown FK and Wiseman JS (1993) Isotope effects and alternative substrate reactivities for tryptophan 2,s-dioxygenase[J]. The Journal of Biological Chemistry 268(24), 17781–17786. 8349662

[52] Li S, Li L, Wu J, Song F, Qin Z, Hou L, Xiao C, Weng J, Qin X and Xu J (2020) TDO promotes hepatocellular carcinoma progression[J]. Oncotargets and Therapy 13, 5845–5855. 32606795 10.2147/OTT.S252929 PMC7311207

[53] Dixon JR, Jung I, Selvaraj S, Shen Y, Antosiewicz-Bourget JE, Lee AY, Ye Z, Kim A, Rajagopal N, Xie W, Diao Y, Liang J, Zhao H, Lobanenkov VV, Ecker JR, Thomson JA and Ren B (2015) Chromatin architecture reorganization during stem cell differentiation[J]. Nature 518(7539), 331–336. 25693564 10.1038/nature14222 PMC4515363

[54] Pallotta MT, Fallarino F, Matino D, Macchiarulo A and AhR-Mediated OC (2014) Non-genomic modulation of IDO1 function[J]. Frontiers in Immunology 5, 497. 25360135 10.3389/fimmu.2014.00497 PMC4197771

[55] Fox E, Oliver T, Rowe M, Thomas S, Zakharia Y, Gilman PB, Muller AJ and Prendergast GC (2018) Indoximod: An immunometabolic adjuvant that empowers T cell activity in cancer[J]. Frontiers in Oncology 8, 370. 30254983 10.3389/fonc.2018.00370 PMC6141803

[56] Awuah SG, Zheng YR, Bruno PM, Hemann MT and Lippard SJ (2015) A platinum(IV) pro-drug preferentially targets indoleamine-2,3-dioxygenase, providing enhanced ovarian cancer immuno-chemotherapy[J]. Journal of the American Chemical Society 137(47), 14854–14857. 26561720 10.1021/jacs.5b10182 PMC4772771

[57] Yue EW, Sparks R, Polam P, Modi D, Douty B, Wayland B, Glass B, Takvorian A, Glenn J, Zhu W, Bower M, Liu X, Leffet L, Wang Q, Bowman KJ, Hansbury MJ, Wei M, Li Y, Wynn R, Burn TC, Koblish HK, Fridman JS, Emm T, Scherle PA, Metcalf B and Combs AP (2017) INCB24360 (Epacadostat), a highly potent and selective indoleamine-2,3-dioxygenase 1 (IDO1) inhibitor for immuno-oncology[J]. ACS Medicinal Chemistry Letters 8(5), 486–491. 28523098 10.1021/acsmedchemlett.6b00391 PMC5430407

[58] Jeffery CJ (2020) Enzymes, pseudoenzymes, and moonlighting proteins: Diversity of function in protein superfamilies[J]. FEBS Journal 287(19), 4141–4149. 32534477 10.1111/febs.15446

[59] Panfili E, Mondanelli G, Orabona C, Gargaro M, Volpi C, Belladonna ML, Rossini S, Suvieri C and Pallotta MT (2023) The catalytic inhibitor epacadostat can affect the non-enzymatic function of IDO1[J]. Frontiers in Immunology 14, 1134551. 37122718 10.3389/fimmu.2023.1134551 PMC10145169

[60] Rossini S, Ambrosino S, Volpi C, Belladonna ML, Pallotta MT, Panfili E, Suvieri C, Macchiarulo A, Mondanelli G and Orabona C (2024) Epacadostat stabilizes the apo-form of IDO1 and signals a pro-tumorigenic pathway in human ovarian cancer cells[J]. Frontiers in Immunology 15, 1346686. 38333210 10.3389/fimmu.2024.1346686 PMC10850306

[61] Dong X, Pan P, Zhang Q, Ye JJ and Zhang XZ (2023) Engineered living bacteriophage-enabled self-Adjuvanting hydrogel for Remodeling tumor microenvironment and cancer therapy[J]. Nano Letters 23(4), 1219–1228. 36729055 10.1021/acs.nanolett.2c04279

[62] Zhu L, Wang X, Ding M, Yu N, Zhang Y, Wu H, Zhang Q, Liu J and Li J (2023) Prodrug-loaded semiconducting polymer hydrogels for deep-tissue sono-immunotherapy of orthotopic glioblastoma[J]. Biomaterials Science 11(20), 6823–6833. 37623749 10.1039/d3bm00585b

[63] Xie Y, Wang M, Qiao L, Qian Y, Xu W, Sun Q, Luo S and Li C (2024) Photothermal-enhanced dual inhibition of lactate/kynurenine metabolism for promoting tumor immunotherapy[J]. Small Methods 8(3), e2300945. 37906051 10.1002/smtd.202300945

[64] Cheng K, Ding Y, Zhao Y, Ye S, Zhao X, Zhang Y, Ji T, Wu H, Wang B, Anderson GJ, Ren L and Nie G (2018) Sequentially responsive therapeutic peptide assembling nanoparticles for dual-targeted cancer immunotherapy[J]. Nano Letters 18(5), 3250–3258. 29683683 10.1021/acs.nanolett.8b01071

[65] Li Y, Wu Y, Fang Z, Zhang Y, Ding H, Ren L, Zhang L, Gong Q, Gu Z and Luo K (2024) Dendritic nanomedicine with boronate bonds for augmented chemo-immunotherapy via synergistic modulation of tumor immune microenvironment[J]. Advanced Materials 36(2), e2307263. 37743633 10.1002/adma.202307263

[66] Xiang D, Han X, Li J, Zhang J, Xiao H, Li T, Zhao X, Xiong H, Xu M and Bi W (2023) Combination of IDO inhibitors and platinum(IV) prodrugs reverses low immune responses to enhance cancer chemotherapy and immunotherapy for osteosarcoma[J]. Materials Today Bio 20, 100675. 10.1016/j.mtbio.2023.100675 PMC10250924 37304579

[67] Liu Y, Xie J, Zhao X, Zhang Y, Zhong Z and Deng C (2022) A polymeric IDO inhibitor based on poly(ethylene glycol)-b-poly(L-tyrosine-co-1-methyl-D-tryptophan) enables facile trident cancer immunotherapy[J]. Biomaterials Science 10(19), 5731–5743. 36039890 10.1039/d2bm01181f

[68] Du W, Chen C, Sun P, Zhang S, Zhang J, Zhang X, Liu Y, Zhang R, Yan C, Fan C, Wu J, Jiang X. Eliciting an immune hot tumor niche with biomimetic drug-based multi-functional nanohybrids augments immune checkpoint blockade-based breast cancer therapy[J]. Nanoscale,2020, 12 (5): 3317–3329. 31976511 10.1039/c9nr09835f

[69] Aitken JB, Austin CJ, Hunt NH, Ball HJ and Lay PA (2014) The Fe-heme structure of met-indoleamine 2,3-dioxygenase-2 determined by X-ray absorption fine structure[J]. Biochemical and Biophysical Research Communications 450(1), 25–29. 24858687 10.1016/j.bbrc.2014.05.054

[70] Austin CJ, Mailu BM, Maghzal GJ, Sanchez-Perez A, Rahlfs S, Zocher K, Yuasa HJ, Arthur JW, Becker K, Stocker R, Hunt NH and Ball HJ (2010) Biochemical characteristics and inhibitor selectivity of mouse indoleamine 2,3-dioxygenase-2[J]. Amino Acids 39(2), 565–578. 20140689 10.1007/s00726-010-0475-9

[71] Mondanelli G, Mandarano M, Belladonna ML, Suvieri C, Pelliccia C, Bellezza G, Sidoni A, Carvalho A, Grohmann U and Volpi C (2021) Current challenges for IDO2 as target in cancer immunotherapy[J]. Frontiers in Immunology 12, 679953. 33968089 10.3389/fimmu.2021.679953 PMC8097162

[72] Merlo LMF, Peng W, Duhadaway JB, Montgomery JD, Prendergast GC, Muller AJ and Mandik-Nayak L (2022) The immunomodulatory enzyme IDO2 mediates autoimmune arthritis through a nonenzymatic mechanism[J]. Journal of Immunology 208(3), 571–581. 10.4049/jimmunol.2100705 PMC8770583 34965962

[73] Li P, Xu W, Liu F, Zhu H, Zhang L, Ding Z, Liang H and Song J (2021) The emerging roles of IDO2 in cancer and its potential as a therapeutic target[J]. Biomedicine and Pharmacotherapy 137, 111295. 33550042 10.1016/j.biopha.2021.111295

[74] Copie-Bergman C, Boulland ML, Dehoulle C, Möller P, Farcet JP, Dyer MJ, Haioun C, Roméo PH, Gaulard P and Leroy K (2003) Interleukin 4-induced gene 1 is activated in primary mediastinal large B-cell lymphoma[J]. Blood 101(7), 2756–2761. 12446450 10.1182/blood-2002-07-2215

[75] Boulland ML, Marquet J, Molinier-Frenkel V, Möller P, Guiter C, Lasoudris F, Copie-Bergman C, Baia M, Gaulard P, Leroy K and Castellano F (2007) Human IL4I1 is a secreted L-phenylalanine oxidase expressed by mature dendritic cells that inhibits T-lymphocyte proliferation[J]. Blood 110(1), 220–227. 17356132 10.1182/blood-2006-07-036210

[76] Zeitler L, Fiore A, Meyer C, Russier M, Zanella G, Suppmann S, Gargaro M, Sidhu SS, Seshagiri S, Ohnmacht C, Köcher T, Fallarino F, Linkermann A and Murray PJ (2021) Anti-ferroptotic mechanism of IL4i1-mediated amino acid metabolism[J]. eLife 10, e64806. 33646117 10.7554/eLife.64806 PMC7946422

[77] Smith JR, Jamie JF and Guillemin GJ (2016) Kynurenine-3-monooxygenase: A review of structure, mechanism, and inhibitors[J]. Drug Discovery Today 21(2), 315–324. 26589832 10.1016/j.drudis.2015.11.001

[78] Kim HT, Na BK, Chung J, Kim S, Kwon SK, Cha H, Son J, Cho JM and Hwang KY (2018) Structural basis for inhibitor-induced hydrogen peroxide production by kynurenine 3-monooxygenase[J]. Cell Chemical Biology 25(4), 426–438, e424. 29429898 10.1016/j.chembiol.2018.01.008

[79] Nevler A, Muller AJ, Sutanto-Ward E, Duhadaway JB, Nagatomo K, Londin E, O’hayer K, Cozzitorto JA, Lavu H, Yeo TP, Curtis M, Villatoro T, Leiby BE, Mandik-Nayak L, Winter JM, Yeo CJ, Prendergast GC and Brody JR (2019) Host IDO2 gene status influences tumor progression and radiotherapy response in KRAS-driven sporadic pancreatic cancers[J]. Clinical Cancer Research 25(2), 724–734. 30266763 10.1158/1078-0432.CCR-18-0814 PMC6335160

[80] He X, He G, Chu Z, Wu H, Wang J, Ge Y, Shen H, Zhang S, Shan J, Peng K, Wei Z, Zou Y, Xu Y and Zhu Q (2021) Discovery of the first potent IDO1/IDO2 dual inhibitors: A promising strategy for cancer immunotherapy[J]. Journal of Medicinal Chemistry 64(24), 17950–17968. 34854662 10.1021/acs.jmedchem.1c01305

[81] Hascitha J, Priya R, Jayavelu S, Dhandapani H, Selvaluxmy G, Sunder Singh S and Rajkumar T (2016) Analysis of kynurenine/tryptophan ratio and expression of IDO1 and 2 mRNA in tumour tissue of cervical cancer patients[J]. Clinical Biochemistry 49(12), 919–924. 27106797 10.1016/j.clinbiochem.2016.04.008

[82] Sadik A, Somarribas Patterson LF, Öztürk S, Mohapatra SR, Panitz V, Secker PF, Pfänder P, Loth S, Salem H, Prentzell MT, Berdel B, Iskar M, Faessler E, Reuter F, Kirst I, Kalter V, Foerster KI, Jäger E, Guevara CR, Sobeh M, Hielscher T, Poschet G, Reinhardt A, Hassel JC, Zapatka M, Hahn U, Von Deimling A, Hopf C, Schlichting R, Escher BI, Burhenne J, Haefeli WE, Ishaque N, Böhme A, Schäuble S, Thedieck K, Trump S, Seiffert M and Opitz CA (2020) IL4I1 is a metabolic immune checkpoint that activates the AHR and promotes tumor progression[J]. Cell 182(5), 1252, e1234–1270. 10.1016/j.cell.2020.07.038 32818467

[83] Zeitler L and Murray PJ (2023) IL4i1 and IDO1: Oxidases that control a tryptophan metabolic nexus in cancer[J]. Journal of Biological Chemistry 299(6), 104827. 37196768 10.1016/j.jbc.2023.104827 PMC10318530

[84] Sabnis RW (2023) Novel IL4I1 inhibitors for treating cancer[J]. ACS Medicinal Chemistry Letters 14(6), 700–701. 37312856 10.1021/acsmedchemlett.3c00180 PMC10258894

[85] Triplett TA, Garrison KC, Marshall N, Donkor M, Blazeck J, Lamb C, Qerqez A, Dekker JD, Tanno Y, Lu WC, Karamitros CS, Ford K, Tan B, Zhang XM, Mcgovern K, Coma S, Kumada Y, Yamany MS, Sentandreu E, Fromm G, Tiziani S, Schreiber TH, Manfredi M, Ehrlich LIR, Stone E and Georgiou G (2018) Reversal of indoleamine 2,3-dioxygenase-mediated cancer immune suppression by systemic kynurenine depletion with a therapeutic enzyme[J]. Nature Biotechnology 36(8), 758–764. 10.1038/nbt.4180 PMC6078800 30010674

[86] Chae SY, Shin H, Woo J, Kang S, Lee SM and Min DH (2024) Metabolic modulation of kynurenine based on Kynureninase-loaded nanoparticle depot overcomes tumor immune evasion in cancer immunotherapy[J]. ACS Applied Materials & Interfaces 16(15), 18490–18502. 38573937 10.1021/acsami.4c00513

[87] Rad Pour S, Morikawa H, Kiani NA, Yang M, Azimi A, Shafi G, Shang M, Baumgartner R, Ketelhuth DFJ, Kamleh MA, Wheelock CE, Lundqvist A, Hansson J and Tegnér J (2019) Exhaustion of CD4+ T-cells mediated by the kynurenine pathway in melanoma[J]. Scientific Reports 9(1), 12150. 31434983 10.1038/s41598-019-48635-x PMC6704156

[88] Kesarwani P, Kant S, Zhao Y, Prabhu A, Buelow KL, Miller CR and Chinnaiyan P (2023) Quinolinate promotes macrophage-induced immune tolerance in glioblastoma through the NMDAR/PPARγ signaling axis[J]. Nature Communications 14(1), 1459. 10.1038/s41467-023-37170-z PMC10020159 36927729

[89] Corsale AM, Di Simone M, Lo Presti E, Picone C, Dieli F and Meraviglia S (2021) Metabolic changes in tumor microenvironment: How could they affect γδ Tcells functions?[J]. Cells 10(11), 2896. 34831116 10.3390/cells10112896 PMC8616133

[90] Stewart BJ, Fergie M, Young MD, Jones C, Sachdeva A, Blain A, Bacon CM, Rand V, Ferdinand JR, James KR, Mahbubani KT, Hook L, Jonas N, Coleman N, Saeb-Parsy K, Collin M, Clatworthy MR, Behjati S and Carey CD (2023) Spatial and molecular profiling of the mononuclear phagocyte network in classic Hodgkin lymphoma[J]. Blood 141(19), 2343–2358. 36758207 10.1182/blood.2022015575

[91] Ait-Belkacem R, Bol V, Hamm G, Schramme F, Van Den Eynde B, Poncelet L, Pamelard F, Stauber J and Gomes B (2017) Microenvironment tumor metabolic interactions highlighted by qMSI: Application to the tryptophan-kynurenine pathway in immuno-oncology[J]. SLAS Discovery 22(10), 1182–1192. 28557618 10.1177/2472555217712659

[92] Sebestyen Z, Prinz I, Dechanet-Merville J, Silva-Santos B and Kuball J (2020) Translating gammadelta (gammadelta) T cells and their receptors into cancer cell therapies[J]. Nature Reviews Drug Discovery 19(3), 169–184. 31492944 10.1038/s41573-019-0038-z

[93] Jonescheit H, Oberg HH, Gonnermann D, Hermes M, Sulaj V, Peters C, Kabelitz D and Wesch D (2020) Influence of indoleamine-2,3-dioxygenase and its metabolite kynurenine on gammadelta Tcell cytotoxicity against ductal pancreatic adenocarcinoma cells[J]. Cells 9(5), 1140. 32384638 10.3390/cells9051140 PMC7290398

[94] Mezrich JD, Fechner JH, Zhang X, Johnson BP, Burlingham WJ and Bradfield CA (2010) An interaction between kynurenine and the aryl hydrocarbon receptor can generate regulatory T cells[J]. Journal of Immunology 185(6), 3190–3198. 10.4049/jimmunol.0903670 PMC2952546 20720200

[95] Caroline Jochems MF and Donahue LM (2016) The IDO1 selective inhibitor epacadostat enhances dendritic cell immunogenicity and lytic ability of tumor antigen-specific T cells[J]. Oncotarget 7(25), 37762. 27192116 10.18632/oncotarget.9326 PMC5122347

[96] Qian F, Liao J, Villella J, Edwards R, Kalinski P, Lele S, Shrikant P and Odunsi K (2012) Effects of 1-methyltryptophan stereoisomers on IDO2 enzyme activity and IDO2-mediated arrest of human T cell proliferation[J]. Cancer Immunology, Immunotherapy 61(11), 2013–2020. 22527253 10.1007/s00262-012-1265-x PMC11028567

[97] De Araujo EF , Loures FV, Preite NW, Feriotti C, Galdino NA, Costa TA, Calich VLG. AhR ligands modulate the differentiation of innate lymphoid cells and t helper cell subsets that control the severity of a pulmonary fungal infection[J]. Frontiers in Immunology,2021, 12: 630938. 33936043 10.3389/fimmu.2021.630938 PMC8085362

[98] Acovic A, Simovic Markovic B, Gazdic M, Arsenijevic A, Jovicic N, Gajovic N, Jovanovic M, Zdravkovic N, Kanjevac T, Harrell CR, Fellabaum C, Dolicanin Z, Djonov V, Arsenijevic N, Lukic ML and Volarevic V (2018) Indoleamine 2,3-dioxygenase-dependent expansion of T-regulatory cells maintains mucosal healing in ulcerative colitis[J]. Therapeutic Advances in Gastroenterology 11, 1–22. 10.1177/1756284818793558 PMC6109841 30159037

[99] Salimi Elizei S, Poormasjedi-Meibod MS, Wang X, Kheirandish M and Ghahary A (2017) Kynurenic acid downregulates IL-17/1L-23 axis in vitro[J]. Molecular and Cellular Biochemistry 431(1-2), 55–65. 28285360 10.1007/s11010-017-2975-3

[100] Swainson LA, Ahn H, Pajanirassa P, Khetarpal V, Deleage C, Estes JD, Hunt PW, Munoz-Sanjuan I and Mccune JM (2019) Kynurenine 3-monooxygenase inhibition during acute simian immunodeficiency virus infection lowers PD-1 expression and improves post-combination antiretroviral therapy CD4^+^ T cell counts and body weight[J]. Journal of Immunology 203(4), 899–910. 10.4049/jimmunol.1801649 PMC6684450 31285277

[101] Zaher SS, Germain C, Fu H, Larkin DF and George AJ (2011) 3-hydroxykynurenine suppresses CD4+ T-cell proliferation, induces T-regulatory-cell development, and prolongs corneal allograft survival[J]. Investigative Ophthalmology and Visual Science 52(5), 2640–2648. 21212175 10.1167/iovs.10-5793 PMC3088555

[102] Bracho-Sanchez E, Hassanzadeh A, Brusko MA, Wallet MA and Keselowsky BG (2019) Dendritic cells treated with exogenous indoleamine 2,3-dioxygenase maintain an immature phenotype and suppress antigen-specific t cell proliferation[J]. International Journal of Fertility & Sterility 5, 100015. 10.1016/j.regen.2019.100015 PMC6884339 31788580

[103] Terness P, Bauer TM, Rose L, Dufter C, Watzlik A, Simon H and Opelz G (2002) Inhibition of allogeneic T cell proliferation by indoleamine 2,3-dioxygenase-expressing dendritic cells: Mediation of suppression by tryptophan metabolites[J]. Journal of Experimental Medicine 196(4), 447–457. 12186837 10.1084/jem.20020052 PMC2196057

[104] Sinclair LV, Neyens D, Ramsay G, Taylor PM and Cantrell DA (2018, 1981) Single cell analysis of kynurenine and system L amino acid transport in T cells[J]. Nature Communications 9(1). 10.1038/s41467-018-04366-7 PMC5958064 29773791

[105] Liu Y, Liang X, Yin X, Lv J, Tang K, Ma J, Ji T, Zhang H, Dong W, Jin X, Chen D, Li Y, Zhang S, Xie HQ, Zhao B, Zhao T, Lu J, Hu ZW, Cao X, Qin FX and Huang B (2017) Blockade of IDO-kynurenine-AhR metabolic circuitry abrogates IFN-gamma-induced immunologic dormancy of tumor-repopulating cells[J]. Nature Communications 8, 15207. 10.1038/ncomms15207 PMC5436221 28488695

[106] Rytelewski M, Meilleur CE, Yekta MA, Szabo PA, Garg N, Schell TD, Jevnikar AM, Sharif S, Singh B and Haeryfar SM (2014) Suppression of immunodominant antitumor and antiviral CD8^+^ T cell responses by indoleamine 2,3-dioxygenase[J]. PLoS One 9(2), e90439. 24587363 10.1371/journal.pone.0090439 PMC3938761

[107] Liu Y, Liang X, Dong W, Fang Y, Lv J, Zhang T, Fiskesund R, Xie J, Liu J, Yin X, Jin X, Chen D, Tang K, Ma J, Zhang H, Yu J, Yan J, Liang H, Mo S, Cheng F, Zhou Y, Zhang H, Wang J, Li J, Chen Y, Cui B, Hu Z-W, Cao X, Xiao-Feng Qin F and Huang B (2018) Tumor-repopulating cells induce PD-1 expression in CD8^+^ T cells by transferring kynurenine and AhR activation[J]. Cancer Cell 33(3), 480–494, e487. 29533786 10.1016/j.ccell.2018.02.005

[108] Shi D, Wu X, Jian Y, Wang J, Huang C, Mo S, Li Y, Li F, Zhang C, Zhang D, Zhang H, Huang H, Chen X, Wang YA, Lin C, Liu G, Song L and Liao W (2022) USP14 promotes tryptophan metabolism and immune suppression by stabilizing IDO1 in colorectal cancer[J]. Nature Communications 13(1), 5644. 10.1038/s41467-022-33285-x PMC9513055 36163134

[109] Malekghasemi S, Majidi J, Baghbanzadeh A, Abdolalizadeh J, Baradaran B and Aghebati-Maleki L (2020) Tumor-associated macrophages: Protumoral macrophages in inflammatory tumor microenvironment[J]. Advanced Pharmaceutical Bulletin 10(4), 556–565. 33062602 10.34172/apb.2020.066 PMC7539304

[110] Vitale I, Manic G, Coussens LM, Kroemer G and Galluzzi L (2019) Macrophages and metabolism in the tumor microenvironment[J]. Cell Metabolism 30(1), 36–50. 31269428 10.1016/j.cmet.2019.06.001

[111] Takenaka MC, Gabriely G, Rothhammer V, Mascanfroni ID, Wheeler MA, Chao CC, Gutierrez-Vazquez C, Kenison J, Tjon EC, Barroso A, Vandeventer T, De Lima KA, Rothweiler S, Mayo L, Ghannam S, Zandee S, Healy L, Sherr D, Farez MF, Prat A, Antel J, Reardon DA, Zhang H, Robson SC, Getz G, Weiner HL and Quintana FJ (2019) Control of tumor-associated macrophages and T cells in glioblastoma via AHR and CD39[J]. Nature Neuroscience 22(5), 729–740. 30962630 10.1038/s41593-019-0370-y PMC8052632

[112] Panitz V, Koncarevic S, Sadik A, Friedel D, Bausbacher T, Trump S, Farztdinov V, Schulz S, Sievers P, Schmidt S, Jurgenson I, Jung S, Kuhn K, Pfluger I, Sharma S, Wick A, Pfander P, Selzer S, Vollmuth P, Sahm F, Von Deimling A, Heiland I, Hopf C, Schulz-Knappe P, Pike I, Platten M, Wick W and Opitz CA (2021) Tryptophan metabolism is inversely regulated in the tumor and blood of patients with glioblastoma[J]. Theranostics 11(19), 9217–9233. 34646367 10.7150/thno.60679 PMC8490504

[113] Yang SL, Tan HX, Niu TT, Liu YK, Gu CJ, Li DJ, Li MQ and Wang HY (2021) The IFN-gamma-IDO1-kynureine pathway-induced autophagy in cervical cancer cell promotes phagocytosis of macrophage[J]. International Journal of Biological Sciences 17(1), 339–352. 33390854 10.7150/ijbs.51241 PMC7757030

[114] Carvajal-Hausdorf DE, Mani N, Velcheti V, Schalper KA and Rimm DL (2017) Objective measurement and clinical significance of IDO1 protein in hormone receptor-positive breast cancer[J]. Journal for Immunotherapy of Cancer 5(1), 81. 29037255 10.1186/s40425-017-0285-7 PMC5644103

[115] Jaufmann J, Lelis FJN, Teschner AC, Fromm K, Rieber N, Hartl D and Beer-Hammer S (2020) Human monocytic myeloid-derived suppressor cells impair B-cell phenotype and function in vitro[J]. European Journal of Immunology 50(1), 33–47. 31557313 10.1002/eji.201948240

[116] Merlo LMF, Peng W and Mandik-Nayak L (2022) Impact of IDO1 and IDO2 on the B cell immune response[J]. Frontiers in Immunology 13, 886225. 35493480 10.3389/fimmu.2022.886225 PMC9043893

[117] Chiesa MD, Carlomagno S, Frumento G, Balsamo M, Cantoni C, Conte R, Moretta L, Moretta A and Vitale M (2006) The tryptophan catabolite l-kynurenine inhibits the surface expression of NKp46- and NKG2D-activating receptors and regulates NK-cell function[J]. Blood 108(13), 4118–4125. 16902152 10.1182/blood-2006-03-006700

[118] Fang X, Guo L, Xing Z, Shi L, Liang H, Li A, Kuang C, Tao B and Yang Q (2022) IDO1 can impair NK cells function against non-small cell lung cancer by downregulation of NKG2D ligand via ADAM10[J]. Pharmacological Research 177, 106132. 35183714 10.1016/j.phrs.2022.106132

[119] Ray A, Song Y and Tai D (2019) Targeting tryptophan catabolic kynurenine pathway enhances antitumor immunity and cytotoxicity in multiple myeloma[J]. Leukemia 34(2), 567–577. 31462737 10.1038/s41375-019-0558-x PMC7132142

[120] Rad Pour S, Morikawa H, Kiani NA, Gomez-Cabrero D, Hayes A, Zheng X, Pernemalm M, Lehtio J, Mole DJ, Hansson J, Eriksson H and Tegner J (2020) Immunometabolic network interactions of the kynurenine pathway in cutaneous malignant melanoma[J]. Frontiers in Oncology 10, 51. 32117720 10.3389/fonc.2020.00051 PMC7017805

[121] Park A, Yang Y, Lee Y, Kim MS, Park YJ, Jung H, Kim TD, Lee HG, Choi I and Yoon SR (2019) Indoleamine-2,3-dioxygenase in thyroid cancer cells suppresses natural killer cell function by inhibiting NKG2D and NKP46 expression via STAT signaling pathways[J]. Journal of Clinical Medicine 8(6), 842. 31212870 10.3390/jcm8060842 PMC6617210

[122] Pal A, Schneider J, Schluter K, Steindorf K, Wiskemann J, Rosenberger F and Zimmer P (2021) Different endurance exercises modulate NK cell cytotoxic and inhibiting receptors[J]. European Journal of Applied Physiology 121(12), 3379–3387. 34477931 10.1007/s00421-021-04735-z PMC8571223

[123] Chaudhary K, Shinde R, Liu H, Gnana-Prakasam JP, Veeranan-Karmegam R, Huang L, Ravishankar B, Bradley J, Kvirkvelia N, Mcmenamin M, Xiao W, Kleven D, Mellor AL, Madaio MP and Mcgaha TL (2015) Amino acid metabolism inhibits antibody-driven kidney injury by inducing autophagy[J]. Journal of Immunology 194(12), 5713–5724. 10.4049/jimmunol.1500277 PMC4458436 25980011

[124] Takamatsu M, Hirata A, Ohtaki H, Hoshi M, Hatano Y, Tomita H, Kuno T, Saito K and Hara A (2013) IDO1 plays an immunosuppressive role in 2,4,6-trinitrobenzene sulfate-induced colitis in mice[J]. Journal of Immunology 191(6), 3057–3064. 10.4049/jimmunol.1203306 23956437

[125] Sittig SP, Van Beek JJP, Florez-Grau G, Weiden J, Buschow SI, Van Der Net MC, Van Slooten R, Verbeek MM, Geurtz PBH, Textor J, Figdor CG, De Vries IJM and Schreibelt G (2021) Human type 1 and type 2 conventional dendritic cells express indoleamine 2,3-dioxygenase 1 with functional effects on T cell priming[J]. European Journal of Immunology 51(6), 1494–1504. 33675038 10.1002/eji.202048580 PMC8251546

[126] Li Q, Harden JL, Anderson CD and Egilmez NK (2016) Tolerogenic phenotype of INF-γ-induced IDO^+^ dendritic cells is maintained via an autocrine IDO1-kynurenine/AhR-IDO loop[J]. Journal of Immunology 197(3), 962–970. 10.4049/jimmunol.1502615 27316681

[127] Zhang L, Huang Y, Cui X, Tan X, Zhu Y, Zhou W, Wang C, Yuan G, Cao Q, Su G, Kijlstra A and Yang P (2020) Increased expression of indoleamine 2,3-dioxygenase in vogt-koyanagi-haradadisease may lead to a shift of Tcell responses toward a treg population[J]. Inflammation 43(5), 1780–1788. 32435912 10.1007/s10753-020-01252-7

[128] Bu L, Baba H, Yoshida N, Miyake K, Yasuda T, Uchihara T, Tan P and Ishimoto T (2019) Biological heterogeneity and versatility of cancer-associated fibroblasts in the tumor microenvironment[J]. Oncogene 38(25), 4887–4901. 30816343 10.1038/s41388-019-0765-y

[129] Kwa MQ, Herum KM and Brakebusch C (2019) Cancer-associated fibroblasts: How do they contribute to metastasis?[J]. Clinical & Experimental Metastasis 36(2), 71–86. 30847799 10.1007/s10585-019-09959-0

[130] Itoh G, Takagane K, Fukushi Y, Kuriyama S, Umakoshi M, Goto A, Yanagihara K, Yashiro M and Tanaka M (2022) Cancer-associated fibroblasts educate normal fibroblasts to facilitate cancer cell spreading and T-cell suppression[J]. Molecular Oncology 16(1), 166–187. 34379869 10.1002/1878-0261.13077 PMC8732346

[131] Chen LB, Zhu SP, Liu TP, Zhao H, Chen PF, Duan YJ and Hu R (2021) Cancer associated fibroblasts promote renal cancer progression through a tdo/kyn/ahr dependent signaling pathway[J]. Frontiers in Oncology 11, 628821. 33842334 10.3389/fonc.2021.628821 PMC8027476

[132] Du R, Zhang X, Lu X, Ma X, Guo X, Shi C, Ren X, Ma X, He Y, Gao Y and Liu Y (2023) PDPN positive CAFs contribute to HER2 positive breast cancer resistance to trastuzumab by inhibiting antibody-dependent NK cell-mediated cytotoxicity[J]. Drug Resistance Updates 68, 100947. 36812747 10.1016/j.drup.2023.100947

[133] Stone TW and Williams RO (2023) Modulation of T cells by tryptophan metabolites in the kynurenine pathway[J]. Trends in Pharmacological Sciences 44(7), 442–456. 37248103 10.1016/j.tips.2023.04.006

[134] Knott PJ and Curzon G (1972) Free tryptophan in plasma and brain tryptophan metabolism[J]. Nature 239(5373), 452–453. 4562870 10.1038/239452a0

[135] Munn DH, Shafizadeh E, Attwood JT, Bondarev I, Pashine A and Mellor AL (1999) Inhibition of T cell proliferation by macrophage tryptophan catabolism[J]. Journal of Experimental Medicine 189(9), 1363–1372. 10224276 10.1084/jem.189.9.1363 PMC2193062

[136] Munn DH and Mellor AL (2016) IDO in the tumor microenvironment: Inflammation, counter-regulation, and tolerance[J]. Trends in Immunology 37(3), 193–207. 26839260 10.1016/j.it.2016.01.002 PMC4916957

[137] Campesato LF, Budhu S, Tchaicha J, Weng CH, Gigoux M, Cohen IJ, Redmond D, Mangarin L, Pourpe S, Liu C, Zappasodi R, Zamarin D, Cavanaugh J, Castro AC, Manfredi MG, Mcgovern K, Merghoub T and Wolchok JD (2020) Blockade of the AHR restricts a Treg-macrophage suppressive axis induced by L-kynurenine[J]. Nature Communications 11(1), 4011. 10.1038/s41467-020-17750-z PMC7419300 32782249

[138] Qu X, Wang Y, Jiang Q, Ren T, Guo C, Hua K and Qiu J (2023) Interactions of Indoleamine 2,3-dioxygenase-expressing LAMP3(+) dendritic cells with CD4(+) regulatory T cells and CD8(+) exhausted T cells: Synergistically remodeling of the immunosuppressive microenvironment in cervical cancer and therapeutic implications[J]. Cancer Communication (London) 43(11), 1207–1228. 10.1002/cac2.12486 PMC10631485 37794698

[139] Xu Y, Zhang H, Sun Q, Geng R, Yuan F, Liu B and Chen Q (2021) Immunomodulatory effects of tryptophan metabolism in the glioma tumor microenvironment[J]. Frontiers in Immunology 12, 730289. 34659216 10.3389/fimmu.2021.730289 PMC8517402

[140] Zhang S, Chen S, Wang Z, Li J, Yuan Y, Feng W, Li W, Chen M and Liu Y (2022) Prognosis prediction and tumor immune microenvironment characterization based on tryptophan metabolism-related genes signature in brain glioma[J]. Frontiers in Pharmacology 13, 1061597. 36386216 10.3389/fphar.2022.1061597 PMC9663932

[141] Li W, Ling L, Xiang L, Ding P and Yue W (2023) Identification and validation of a risk model and molecular subtypes based on tryptophan metabolism-related genes to predict the clinical prognosis and tumor immune microenvironment in lower-grade glioma[J]. Frontiers in Cellular Neuroscience 17, 1146686. 36925967 10.3389/fncel.2023.1146686 PMC10011102

[142] Yan J, Chen D, Ye Z, Zhu X, Li X, Jiao H, Duan M, Zhang C, Cheng J, Xu L, Li H and Yan D (2024) Molecular mechanisms and therapeutic significance of tryptophan metabolism and signaling in cancer[J]. Molecular Cancer 23(1), 241. 39472902 10.1186/s12943-024-02164-y PMC11523861

[143] Frankel TL and Pasca Di Magliano M (2022) Immune sensing of microbial metabolites: Action at the tumor[J]. Immunity 55(2), 192–194. 35139348 10.1016/j.immuni.2022.01.009

[144] Bender MJ, Mcpherson AC, Phelps CM, Pandey SP, Laughlin CR, Shapira JH, Medina Sanchez L, Rana M, Richie TG, Mims TS, Gocher-Demske AM, Cervantes-Barragan L, Mullett SJ, Gelhaus SL, Bruno TC, Cannon N, Mcculloch JA, Vignali DAA, Hinterleitner R, Joglekar AV, Pierre JF, Lee STM, Davar D, Zarour HM and Meisel M (2023) Dietary tryptophan metabolite released by intratumoral lactobacillus reuteri facilitates immune checkpoint inhibitor treatment[J]. Cell 186(9), 1846, e1826–1862. 10.1016/j.cell.2023.03.011 PMC10148916 37028428

[145] Hezaveh K, Shinde RS, Klötgen A, Halaby MJ, Lamorte S, Ciudad MT, Quevedo R, Neufeld L, Liu ZQ, Jin R, Grünwald BT, Foerster EG, Chaharlangi D, Guo M, Makhijani P, Zhang X, Pugh TJ, Pinto DM, Co IL, Mcguigan AP, Jang GH, Khokha R, Ohashi PS, O’kane GM, Gallinger S, Navarre WW, Maughan H, Philpott DJ, Brooks DG and Mcgaha TL (2022) Tryptophan-derived microbial metabolites activate the aryl hydrocarbon receptor in tumor-associated macrophages to suppress anti-tumor immunity[J]. Immunity 55(2), 324, e328–340. 10.1016/j.immuni.2022.01.006 PMC8888129 35139353

[146] Kuan YD and Lee CH (2016) Salmonella overcomes tumor immune tolerance by inhibition of tumor indoleamine 2, 3-dioxygenase 1 expression[J]. Oncotarget 7(1), 374–385. 26517244 10.18632/oncotarget.6258 PMC4808005

[147] Schroten H, Spors B, Hucke C, Stins M, Kim KS, Adam R and Däubener W (2001) Potential role of human brain microvascular endothelial cells in the pathogenesis of brain abscess: Inhibition of Staphylococcus aureus by activation of indoleamine 2,3-dioxygenase[J]. Neuropediatrics 32(4), 206–210. 11571701 10.1055/s-2001-17375

[148] Labadie BW, Bao R and Luke JJ (2019) Reimagining IDO pathway inhibition in cancer immunotherapy via downstream focus on the tryptophan-kynurenine-aryl hydrocarbon axis[J]. Clinical Cancer Research 25(5), 1462–1471. 30377198 10.1158/1078-0432.CCR-18-2882 PMC6397695

[149] Kober C, Roewe J, Schmees N, Roese L, Roehn U, Bader B, Stoeckigt D, Prinz F, Gorjánácz M, Roider HG, Olesch C, Leder G, Irlbacher H, Lesche R, Lefranc J, Oezcan-Wahlbrink M, Batra AS, Elmadany N, Carretero R, Sahm K, Oezen I, Cichon F, Baumann D, Sadik A, Opitz CA, Weinmann H, Hartung IV, Kreft B, Offringa R, Platten M and Gutcher I (2023) Targeting the aryl hydrocarbon receptor (AhR) with BAY 2416964: A selective small molecule inhibitor for cancer immunotherapy[J]. Journal for Immunotherapy of Cancer 11(11), e007495. 37963637 10.1136/jitc-2023-007495 PMC10649913

[150] Moyer BJ, Rojas IY, Murray IA, Lee S, Hazlett HF, Perdew GH and Tomlinson CR (2017) Indoleamine 2,3-dioxygenase 1 (IDO1) inhibitors activate the aryl hydrocarbon receptor[J]. Toxicology and Applied Pharmacology 323, 74–80. 28336214 10.1016/j.taap.2017.03.012 PMC5495139

[151] Wyatt M and Greathouse KL (2021) Targeting dietary and microbial tryptophan-indole metabolism as therapeutic approaches to colon cancer[J]. Nutrients 13(4), 1189. 33916690 10.3390/nu13041189 PMC8066279

[152] Zhang J, Liu Y, Zhi X, Xu L, Tao J, Cui D and Liu TF (2024) Tryptophan catabolism via the kynurenine pathway regulates infection and inflammation: From mechanisms to biomarkers and therapies[J]. Inflammation Research 73(6), 979–996. 38592457 10.1007/s00011-024-01878-5

[153] Odunsi K, Qian F, Lugade AA, Yu H, Geller MA, Fling SP, Kaiser JC, Lacroix AM, D’amico L, Ramchurren N, Morishima C, Disis ML, Dennis L, Danaher P, Warren S, Nguyen VA, Ravi S, Tsuji T, Rosario S, Zha W, Hutson A, Liu S, Lele S, Zsiros E, Mcgray AJR, Chiello J, Koya R, Chodon T, Morrison CD, Putluri V, Putluri N, Mager DE, Gunawan R, Cheever MA, Battaglia S and Matsuzaki J (2022) Metabolic adaptation of ovarian tumors in patients treated with an IDO1 inhibitor constrains antitumor immune responses[J]. Science Translational Medicine 14(636), eabg8402. 35294258 10.1126/scitranslmed.abg8402 PMC9311231

[154] Song J, Cheng M, Xie Y, Li K and Zang X (2023) Efficient tumor synergistic chemoimmunotherapy by self-augmented ROS-responsive immunomodulatory polymeric nanodrug[J]. Journal of Nanobiotechnology 21(1), 93. 36927803 10.1186/s12951-023-01842-1 PMC10018933

[155] Nguyen TT, Shin DH, Sohoni S, Singh SK, Rivera-Molina Y, Jiang H, Fan X, Gumin J, Lang FF, Alvarez-Breckenridge C, Godoy-Vitorino F, Zhu L, Zheng WJ, Zhai L, Ladomersky E, Lauing KL, Alonso MM, Wainwright DA, Gomez-Manzano C and Fueyo J (2022) Reshaping the tumor microenvironment with oncolytic viruses, positive regulation of the immune synapse, and blockade of the immunosuppressive oncometabolic circuitry[J]. Journal for Immunotherapy of Cancer 10(7), e004935. 35902132 10.1136/jitc-2022-004935 PMC9341188

[156] Kelly CM, Qin LX, Whiting KA, Richards AL, Avutu V, Chan JE, Chi P, Dickson MA, Gounder MM, Keohan ML, Movva S, Nacev BA, Rosenbaum E, Adamson T, Singer S, Bartlett EK, Crago AM, Yoon SS, Hwang S, Erinjeri JP, Antonescu CR, Tap WD and D’angelo SP (2023) A phase II study of epacadostat and pembrolizumab in patients with advanced sarcoma[J]. Clinical Cancer Research 29(11), 2043–2051. 36971773 10.1158/1078-0432.CCR-22-3911 PMC10752758

[157] Cho BC, Braña I, Cirauqui B, Aksoy S, Couture F, Hong RL, Miller WH, Chaves-Conde M, Teixeira M, Leopold L, Munteanu M, Ge JY, Swaby RF and Hughes BGM (2024) Pembrolizumab plus epacadostat in patients with recurrent/metastatic head and neck squamous cell carcinoma (KEYNOTE-669/ECHO-304): A phase 3, randomized, open-label study[J]. BMC Cancer 23(Suppl 1), 1254. 39054467 10.1186/s12885-023-11316-0 PMC11270762

[158] Fujiwara Y, Kato S, Nesline MK, Conroy JM, Depietro P, Pabla S and Kurzrock R (2022) Indoleamine 2,3-dioxygenase (IDO) inhibitors and cancer immunotherapy[J]. Cancer Treatment Reviews 110, 102461. 36058143 10.1016/j.ctrv.2022.102461 PMC12187009

[159] Huang Q, Xia J, Wang L, Wang X, Ma X, Deng Q, Lu Y, Kumar M, Zhou Z, Li L, Zeng Z, Young KH, Yi Q, Zhang M and Li Y (2018) miR-153 suppresses IDO1 expression and enhances CAR T cell immunotherapy[J]. Journal of Hematology & Oncology 11(1), 58. 29685162 10.1186/s13045-018-0600-x PMC5914051

[160] Caforio M, Sorino C, Caruana I, Weber G, Camera A, Cifaldi L, De Angelis B, Del Bufalo F, Vitale A, Goffredo BM, De Vito R, Fruci D, Quintarelli C, Fanciulli M, Locatelli F and Folgiero V (2021) GD2 redirected CAR T and activated NK-cell-mediated secretion of IFNγ overcomes MYCN-dependent IDO1 inhibition, contributing to neuroblastoma cell immune escape[J]. Journal for Immunotherapy of Cancer 9(3), e001502. 33737337 10.1136/jitc-2020-001502 PMC7978286

[161] Su Q, Wang C, Song H, Zhang C, Liu J, Huang P, Zhang Y, Zhang J and Wang W (2021) Co-delivery of anionic epitope/CpG vaccine and IDO inhibitor by self-assembled cationic liposomes for combination melanoma immunotherapy[J]. Journal of Materials Chemistry B 9(18), 3892–3899. 33928989 10.1039/d1tb00256b

[162] Andersen MH (2019) The T-win® technology: Immune-modulating vaccines[J]. Seminars in Immunopathology 41(1), 87–95. 29968045 10.1007/s00281-018-0695-8

